# Horizontally Acquired Glycosyltransferase Operons Drive Salmonellae Lipopolysaccharide Diversity

**DOI:** 10.1371/journal.pgen.1003568

**Published:** 2013-06-20

**Authors:** Mark R. Davies, Sarah E. Broadbent, Simon R. Harris, Nicholas R. Thomson, Marjan W. van der Woude

**Affiliations:** 1 Centre for Immunology and Infection, Hull York Medical School and the Department of Biology, University of York, York, United Kingdom; 2 The Wellcome Trust Sanger Institute, The Wellcome Trust Genome Campus, Hinxton, Cambridgeshire, United Kingdom; Uppsala University, Sweden

## Abstract

The immunodominant lipopolysaccharide is a key antigenic factor for Gram-negative pathogens such as salmonellae where it plays key roles in host adaptation, virulence, immune evasion, and persistence. Variation in the lipopolysaccharide is also the major differentiating factor that is used to classify *Salmonella* into over 2600 serovars as part of the Kaufmann-White scheme. While lipopolysaccharide diversity is generally associated with sequence variation in the lipopolysaccharide biosynthesis operon, extraneous genetic factors such as those encoded by the glucosyltransferase (*gtr*) operons provide further structural heterogeneity by adding additional sugars onto the O-antigen component of the lipopolysaccharide. Here we identify and examine the O-antigen modifying glucosyltransferase genes from the genomes of *Salmonella enterica* and *Salmonella bongori* serovars. We show that *Salmonella* generally carries between 1 and 4 *gtr* operons that we have classified into 10 families on the basis of *gtrC* sequence with apparent O-antigen modification detected for five of these families. The *gtr* operons localize to bacteriophage-associated genomic regions and exhibit a dynamic evolutionary history driven by recombination and gene shuffling events leading to new gene combinations. Furthermore, evidence of Dam- and OxyR-dependent phase variation of *gtr* gene expression was identified within eight *gtr* families. Thus, as O-antigen modification generates significant intra- and inter-strain phenotypic diversity, *gtr*-mediated modification is fundamental in assessing *Salmonella* strain variability. This will inform appropriate vaccine and diagnostic approaches, in addition to contributing to our understanding of host-pathogen interactions.

## Author Summary

Bacterial pathogens frequently evolve mechanisms to vary the composition of their surface structures. The consequence is enhanced long-term survival by facilitating persistence and evasion of the host immune system. *Salmonella* sp., cause severe infections in a range of mammalian hosts and guard themselves with a protective coat, termed the O-antigen. Through genome sequence analyses we found that *Salmonella* have acquired an unprecedented repertoire of genetic sequences for modifying their O-antigen coat. There is strong evidence that these genetic factors have a dynamic evolutionary history and are spread through the bacterial population by bacteriophage. In addition to this genetic repertoire, we determined that *Salmonella* can and often do employ stochastic mechanisms for expression of these genetic factors. This means that O-antigen coat diversity can be generated within a *Salmonella* population that otherwise has a common genome. Our data significantly enhance our appreciation of the genetic and regulatory characteristics underpinning *Salmonella* O-antigen diversity. The role attributed to bacteriophage in generating this diversity highlights that *Salmonella* are acquiring an extensive repertoire of O-antigen modifying traits that may enhance the pathogen's ability to persist and cause disease in mammalian hosts. Such genetic traits may make useful markers for defining new epidemiological and diagnostic tools.

## Introduction

There are two recognized *Salmonella* species, *S. bongori* and *S. enterica*, which can be divided into ∼2600 recognized *Salmonella* serovars according to the Kaufmann-White-Le Minor (KW) classification scheme [1], [2]. Over half of these serovars are represented by *S. enterica* subspecies *enterica* (*S. enterica* subspecies I), which constitute 99% of human clinical *Salmonella* infections. The KW Scheme is governed by differences in antigenicity of the O-antigen of the lipopolysaccharide (LPS) and the flagella (H factor). The KW O-serotype is dependent on the reactivity of immune sera against immunodominant epitopes of the O-antigen, which in turn is dictated by the O-antigen structure. The KW *Salmonella* classification scheme is based on a panel of antisera recognizing 58 different O-antigen epitopes that allows classification of *Salmonella* into 46 different O-serogroups with some serogroups having multiple O-antigen epitopes [1], [2]. More than 58 epitopes have been identified, but are not employed for classification purposes within the current scheme.

O-antigen diversity derives largely from differences in the carbohydrate composition and structure of the basal units of the O-antigen. This has been attributed to horizontal gene transfer events within the O-antigen biosynthesis locus, *rfb* (reviewed by [3], [4]). Further O-antigen structural and antigenic modifications have been identified that are driven by genes residing outside of the *rfb* loci such as the O-acetyltransferase gene, *oafA* that modifies the serotype through acetylation [5], *wzy*-like genes that alter linkages between O-antigen units [6] and glycosyltransferase (*gtr*) operons that add sugars onto the basic O-antigen structure thus changing the O-serotype [7]–[11].

The best studied example of O-antigen glucosylation in *Salmonella* is due to bacteriophage P22 lysogenization of *S. enterica* subsp. *enterica* serovar Typhimurium (*S.* Typhimurium) strains. Phage P22 encodes a *gtr* operon, formerly designated as “*con*” or “*a1*”, that results in the addition of glucose to the galactose moiety of the O-antigen basal unit by a 1–6 linkage, leading to seroconversion and the additional recognition by O:1 typing sera [8]–[10]. A second *gtr* cluster has been identified in the chromosome of *S.* Typhimurium [7]. Formerly called “*oafR*” [12], this *gtr* operon glucosylates the same galactose residue as the P22 *gtr* operon, but with a different (1–4) linkage [7]. This modification yields a change from O:12 to sub-type O:12_2_ serotype. However, this ‘sub-type’ change is not identified as part of standard *Salmonella* serotyping carried out in centers using the KW classification scheme. Furthermore, according to the KW classification scheme, both O:1 and O:12_2_ expressing *S.* Typhimurium strains are collectively designated as the same serogroup, serogroup B. Thus, *Salmonella* strains that are identified as having the same clinical O-serogroup or O-serotype may in fact differ in their detailed O-antigen structures.

The *gtr* gene clusters described above consist of three genes, referred to in *Salmonella* as *gtrABC*. *Shigella* have similar clusters, and combining genetic and biochemical analysis for both species, a model has been proposed for the role of the three gene products (reviewed by [13]). GtrA and GtrB are proposed to be the bactoprenol-linked glucosyltranslocase or ‘flippase’ and the bactoprenol glucosyltransferase respectively, while GtrC is defined as the serotype-specific glycosyltransferase. There is little sequence homology between the known GtrC sequences [11], [13], probably reflecting the variety of O-antigen substrates recognized and the differing enzymatic activities.

The role of the O-antigen for the biology of pathogens is diverse and not fully understood. The O-antigen may be required for virulence as is the case for *Escherichia coli*, whereas other pathogens can succeed in the absence of an O-antigen. In general, the composition, structure and length of the O-antigen as determined by the *rfb* cluster, will influence antigenicity and can affect direct interactions with host cells, facilitate molecular mimicry, and alter the efficacy of the innate immune response [14], [15]. In salmonellae, the composition may contribute to persistence and sequential infection of serovars [16]. O-antigen diversity in *Salmonella* has also been implicated in host adaptation by altering susceptibility to digestion by intestinal amoebae [17], [18].

Likewise, the importance of O-antigen modification as mediated by non-*rfb* genetic mechanisms such as *gtr* and *oafA* remains to be fully elucidated, but studies indicate an impact on various aspects of *Salmonella* biology. O-antigen modification can alter phage susceptibility when the O-antigen is a primary or secondary receptor, with very specific modifications affecting specific phage. This was shown recently in *S.* Typhimurium, with expression of the P22 *gtrABC* decreasing P22 infectivity [19]. In addition, the phase variable expression of the P22 *gtr* operon [20] confers transient resistance to phage SPC35 [19]. The immune response to the O-antigen is also a key factor in determining spread of *Salmonella* and therefore, immune evasion or ‘sero-conversion’ as a result of changes in antigenicity due to modification of the O-antigen, may contribute to *Salmonella* persistence and dissemination [16], [21], [22]. Furthermore, O-antigen modification may have a role in gut persistence in a mouse model of *S.* Typhimurium infection [7]. The general effects associated with *rfb*-dependent composition may also be affected by O-antigen modification, but this has received little attention to date.

In contrast to the large body of work detailing the biochemical diversity and serotypic association of *Salmonella* O-antigens, relatively little insight has been accumulated regarding the genetic systems that promote *Salmonella* O-antigen heterogeneity. Here we explore the *gtr* repertoire in salmonellae based on genome sequence analysis and provide initial characterization to further our understanding of the role of these gene clusters in shaping the evolution, transmission and virulence of this important pathogen.

## Results

### High prevalence of*gtr*-like operons within *Salmonella* serovars that cluster into 10 distinct GtrC ‘families’

BLASTn was used with phage P22 *gtrA*, *gtrB* and *gtrC* genes as query sequences to identify *gtr*-like operons in 57 *Salmonella* genomes constituting the two known *Salmonella* species, *S. enterica* (n = 28) and *S. bongori* (n = 29) and four O-antigen modifying *Salmonella* bacteriophage genomes (P22, ST104, ST64T and epsilon34). Based on this analysis, a total of 59 *gtr*-like operons were identified: 52 in the 22 *S. enterica* subspecies *enterica* (subspecies I) genomes, 3 in 3 *S. bongori* serovars and 4 in the 4 *Salmonella* bacteriophage genomes (Tables S1 and S2). No *gtr* operons were identified in the singular genome sequences representing *S. enterica* subspecies *salamae* (subspecies II); *arizonae* (subspecies IIIa); *diarizonae* (subspecies IIIb); *houtenae* (subspecies IV) and *indica* (subspecies VI). While the three *S. bongori* genomes harbored a single *gtr* operon, 20 of the 22 *S. enterica* subspecies I genomes carried between two and four different *gtr* operons (Table S1). Thus, despite the important role that O-antigen structure plays in assigning the serogroup according to the Kaufmann-White *Salmonella* classification scheme, this genomic repertoire of *gtr* operons might suggest the existence of a level of antigenic complexity that is not defined in the standard typing scheme.

**Figure 1:**
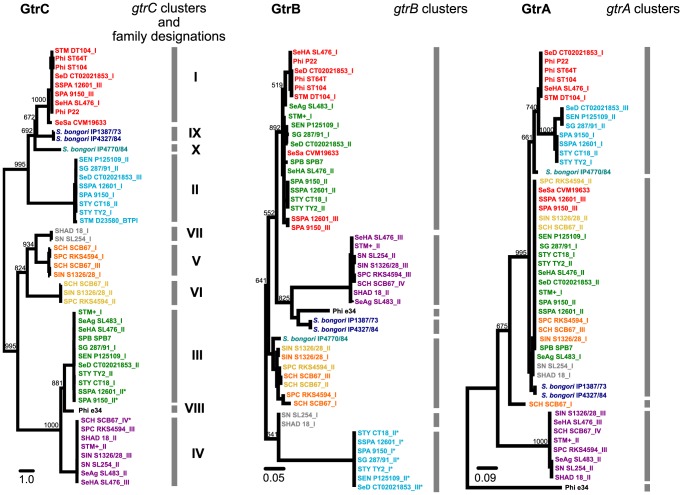
Phylogenetic and clustering analyses of the GtrC, GtrB and GtrA gene products from*Salmonella*. A total of 59 *gtr*-like operons were identified from BLAST analysis of 22 complete *S. enterica* subspecies I complete genome sequences, 28 draft *S. bongori* genome sequences and 4 phage genome sequences. The grey bar indicates clusters of sequences that segregated on the basis of genomic sequence as determined by the Bayesian algorithm software, BAPS [23], [24]. 10 GtrC clusters (‘GtrC families’) are identified by clustering and colored accordingly. This GtrC family color scheme is applied to the GtrB and GtrA trees to examine evolutionary relationships between the 3 gene products. GtrB and GtrA segregate into 7 and 5 clusters accordingly and the overall clusters are more closely related compared to GtrC gene products (scale bar). The lack of congruent clustering between GtrC, GtrB and GtrA gene products reflect different evolution histories of the gene products. Maximum likelihood rectangular phylograms were derived from ClustalW2 alignments using PhyML. Horizontal distances are proportional to sequence differences per site relative to the scalebar shown for each tree. Bootstrap values, out of 1000 trials, are shown on the tree branches. Each terminal node is labeled with a Gtr acronym assigned in Table S1. Asterisk denotes sequences predicted to contain deletions or frameshift mutations. The six *S.* Typhimurium genome sequences examined in this study (Table S1) share 100% sequence identity in their family III and family IV GtrC sequences and are collectively represented in this figure as STM_I and STM_II respectively.

Since the *gtrC* gene product is purported to be responsible for the serotype-specific modification of the O-antigen, this gene product was used to define the functional relationships of the 59 *gtrABC* operons. Maximum likelihood approaches and Bayesian clustering analyses were used to plot the genetic relationships of all 59 GtrC products included in this study [23], [24]. Figure 1 shows that the 59 GtrC's form 10 distinct clusters, herein defined as ‘GtrC families’ (Figure 1, ‘GtrC’). Phylogenetic analyses of the cognate GtrA and GtrB gene products identified 5 and 7 clusters respectively (Figure 1, ‘GtrA’ and ‘GtrB’). Only gene clusters represented by GtrC families IV and VIII were also represented in the same, single *gtrA*, *gtrB* cluster (Figure 1). In general, sequence conservation between any two GtrC families was very low. For example, the GtrC of phage P22, *gtr* ^P22^ (GtrC family I) and *S.* Typhimurium *gtr* ^LT2_I^ (GtrC family III) share less than 18% amino acid identity, yet both add a glucose residue to the same galactose moiety of the *S.* Typhimurium O-antigen but via different linkages [7]–[10]. In contrast, orthologous GtrC proteins within the same GtrC family generally exhibit greater than 96% amino acid identity. Unlike the 10 GtrC families, the amino acid identity between any two GtrA and GtrB families are much higher, 84% and 90% respectively. Conserved internal gene deletions were found in the *gtrB* gene of all family II *gtr* operons and premature stop codons are predicted to occur within three GtrC gene products: One from GtrC family IV (*S.* Choleraesuis *gtrC* ^SCB67_IV^) and two from GtrC family III (*S.* Paratyphi *gtrC* ^9150_II^ and *S.* Paratyphi *gtrC* ^12601_II^).

### Dynamic and ongoing evolutionary history of the*gtr* operon

**Figure 2:**
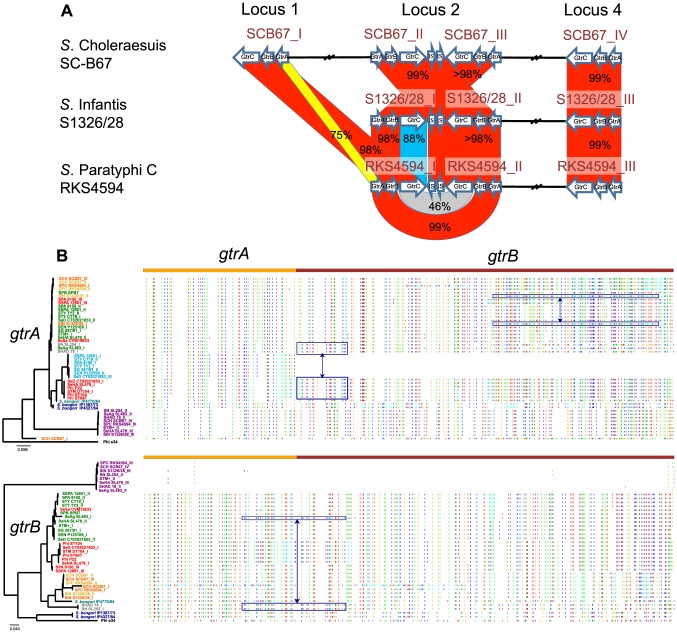
Extensive recombination within*gtr* operons. A) Schematic representation of a BLAST comparison between the *gtr* operons from three Kaufmann-White serogroup C_1_ genomes; *S.* Paratyphi C, *S.* Infantis and *S.* Choleraesuis. Red shaded regions indicate gene products with >98% protein similarity; blue regions <90% similarity; yellow regions <80% similarity and Grey regions <50% similarity as determined by ClustalW2 amino acid alignments [42]. A possible *gtr* recombination is evident between the *gtrA* or *gtrBC* of *S.* Choleraesuis *gtr* ^SCB67_I^ and the *gtrA* or *gtrBC* of *S.* Paratyphi C *gtr* ^RKS4594_I^ *gtr* operons. *S.* Choleraesuis *gtr* ^SCB67_I^ is encompassed within a full-length P22-like prophage termed Scho1 [11]. Genome integration sites as depicted in Figure 3 are represented above the Figure. IS refers to insertion-like sequences. B) Mapping of homoplasic SNPs within *gtrA* and *gtrB* gene sequences. On the left of the figure is the mid-point rooted phylogeny of the *gtrA* and *gtrB* gene sequences with gene names colored according to GtrC Family designation (Figure 1). Colored vertical lines depict homoplasic bases (red:A, blue:T, green:C, orange:G) that differ from the ancestral sequence and are shown relative to the concatenated *gtrAB* gene sequence shown on top. The pattern of lines represents a homoplasic ‘barcode’ of similarity between strains and is used to indicate recombined regions. High homoplasic SNP density across both *gtrA* and *gtrB* genes indicates a dynamic and complex evolutionary history driven by extensive recombination. Some examples of recombined regions are indicated by blue boxes. In the case of *S.* Agona *gtr* ^SL483_I^ and *S.* Hadar *gtr* ^18_I^/*S.* Newport *gtr* ^SL254_I^, recombination is not restricted by *gtrAB* gene boundaries.

The general lack of congruency between *gtrA*, *gtrB* and *gtrC* clustering (Figure 1) raises the possibility that recombination has shaped the evolutionary history of the *gtr* operon. Two approaches were employed to investigate this further. Comparative alignments of the three KW serogroup C_1_ genomes *S.* Paratyphi C, *S.* Choleraesuis and *S.* Infantis, showed that *S.* Choleraesuis possessed an extra *gtr* operon, *gtr* ^SCB67_I^ (Figure 2A). While the GtrB and GtrC proteins of *S.* Choleraesuis *gtr* ^SCB67_I^ exhibited over 98 percent amino acid identity to the GtrB and GtrC proteins of *S.* Paratyphi C *gtr* ^RKS4594_I^, the GtrA protein of these operons were substantially lower at 75 percent (Figure 2A). These data suggest that allelic exchange may have occurred between the *gtrA* or *gtrBC* of a *gtr* ^SCB67_I^-like and *gtr* ^RKS4594_I^-like progenitor. A second approach using homoplasic SNPs as a marker of recombination identified extensive clusters of homoplasies scattered throughout the *gtrA* and *gtrB* sequences (Figure 2B). In the case of *S.* Agona *gtr* ^SeAg_I^ and *S.* Hadar *gtr* ^18_I^ the recombination event was not restricted by gene boundaries. These data illustrate a dynamic and complex picture of ongoing *gtr* evolution across *Salmonella* genomes and raise the possibility that a degree of functional redundancy may exist in the Gtr system with new combinations of *gtrA*, *gtrB* and *gtrC* sequences arising through recombination.

### Gtr families localize to four genomic locations within*Salmonella* serovars

**Figure 3:**
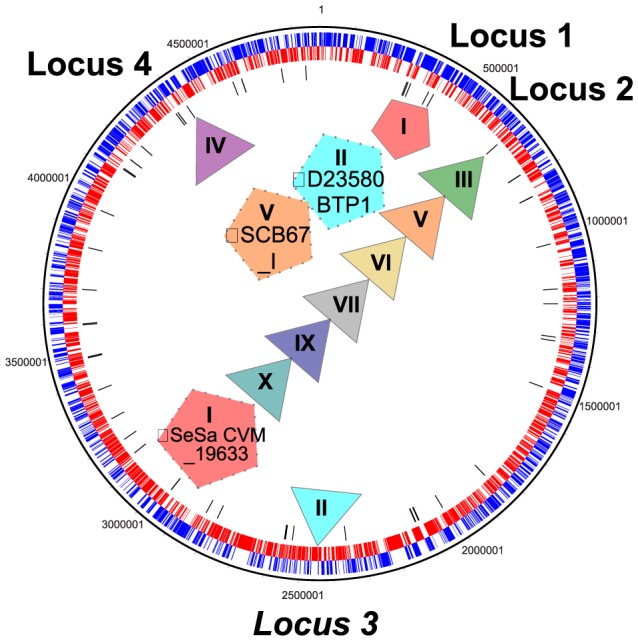
Genome localization of*Salmonella gtr* operons. Circular representation of the serogroup B *S.* Typhimurium D23580 genome (accession number FN424405) indicating that 9 GtrC families localize to four genomic regions (locus 1–4) relative to the D23580 genome. The italicized locus 3 (SPI-17) site is not represented in KW serogroup B genomes and thus, its relative location in *S.* Typhimurium D23580 is shown. Triangular *gtr* operons point the common location of GtrC family members. Pentagonal *gtr* operons are associated with full-length prophage. Dotted pentagonal operons with the phi symbol ‘Φ’ indicate *gtr* operons associated with full-length prophage that do not localize with their ‘parent’ GtrC family members. Gtr acronyms for these three *gtr* operons are shown (Table S1). Color code of the pentagon/triangles relate to the GtrC family clustering defined in Figure 1. The circular *S.* Typhimurium D23580 genome depicts forward open reading frames in blue, reverse open reading frames in red and tRNA in black.

To determine whether *gtr* operons localize to defined genomic loci, 55 *gtr* operons representing 9 of the 10 *Salmonella* GtrC families were mapped onto the *S.* Typhimurium strain D23580 genomic backbone to show relative locations. All *gtr* operons identified in this study localized to four genomic regions (Figure 3, locus 1 to 4). These four regions are localized alongside tRNA genes and constitute the P22 (locus 1) and P2 (locus 4) integration sites, and the stably maintained phage derived genomic regions SPI-16 (locus 2) and SPI-17 (locus 3) [25]. While locus 3 and 4 contain a single GtrC family (GtrC family II and IV respectively), locus 1 and 2 harbor representatives of three and seven different GtrC families, respectively (Figure 3).

**Figure 4:**
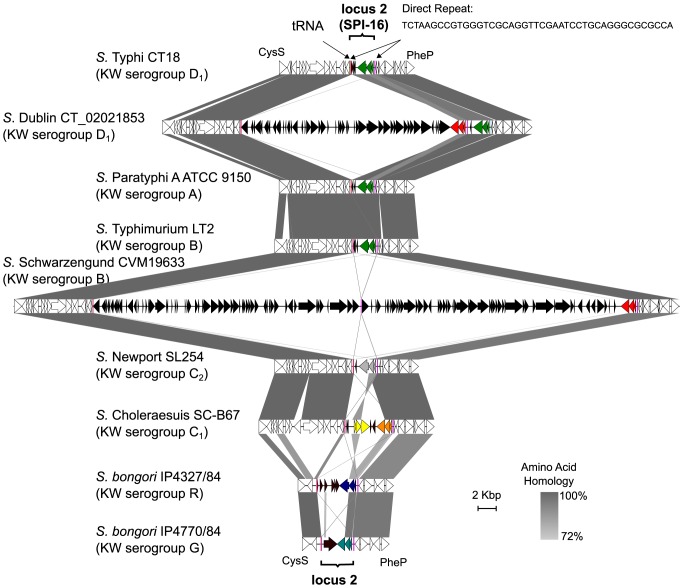
Heterogeneity of Gtr families localized within locus 2 of*Salmonella* serovars. Represented is an alignment and sequence comparison of the *cysS* to *pheP* genomic region encompassing locus 2 (SPI-16, Figure 3) from nine *gtr* positive *Salmonella* serovars. The phage-associated locus 2 is defined by a pair of direct repeats (pink) and a 5′ Arg-tRNA [25]. Black coding sequences refer to those located within locus 2 boundaries, while white coding sequences are located outside locus 2. The *gtr* operon located within locus 2 is colored on the basis of GtrC family clustering (Figure 1). A different colored GtrC family likely reflects a functionally different *gtr* operon. *S.* Schwarzengrund contains a *gtr* positive lambda-like prophage and *S.* Dublin harbors a large remnant P22-like phage [11] within locus 2. Regions of genetic similarity between genome sequences are shaded in grey and were determined by BLASTn analysis using Easyfig [52].

Four *gtr* operons, *S.* Schwarzengrund *gtr* ^CVM19633^ (family I), *S.* Dublin *gtr* ^CT02021853_I^ (family I), *S.* Typhimurium *gtr* ^D23580_BTP1^ (family II), and *S.* Choleraesuis *gtr* ^SCB67_I^ (family V), did not localize to the same genomic location as their other family members (Figure 3). All four of these operons appear to be located on full-length prophage (Figure 3). This is consistent with lysogenic phage carrying *gtr* operons having alternative site-specific integration sites in the *Salmonella* genome. Indeed, the P22-like GtrC family, family I, is inserted at the P22 attachment site (locus 1). In contrast, a lambda-like prophage from *S.* Schwarzengrund CVM19633 harboring the family I GtrC, GtrC^CVM19633^ and a P22-like prophage from *S.* Dublin carrying two *gtr* operons, GtrC^CT02021853_I^ (family I) and GtrC^CT02021853_II^ (GtrC) are integrated at locus 2 (Figures 3, 4), which is also the integration site for the GtrC family I and III operons carried on a novel P22-like prophage [11].

### The correlation between GtrC repertoire and serotype

The presence of a specific GtrC family member at locus 2 (Figures 3, 4) appears to correlate with the O-antigen substrate of the host genome. For example, in Kaufmann-White serogroup A, B and D_1_ genomes, which share a common tri-saccharide O-antigen backbone [7], [26], locus 2 contained a GtrC family III operon. In contrast, the same locus 2 site in KW serogroup C_1_ serovars and C_2_ were occupied by two adjacent *gtr* operons encoding GtrC family V and VI members, and a family VII GtrC, respectively (Figure 4). The absence of a family III GtrC, in serogroups C_1_ and C_2_ is consistent with the absence of the galactose moiety in the O-antigen that is the receptor for the family III GtrC [26].

Overall however, the repertoire of *gtr* operons did not correlate with the Kaufmann-White O-serogroupings. For example, serovars represented by serogroups B, C_1_ and D_1_ possessed different numbers of *gtr* operons (Table S1). Furthermore, different strains of a single serovar can encode different numbers of *gtr* operons. For example, *S.* Typhimurium strains LT2, DT2, SL1344 and 14028s have two *gtr* operons compared to *S.* Typhimurium strains DT104 and D23580, which possess three *gtr* operons (Table S1).

In combination with the recombinogenic signature of genes within the *gtr* operons, these data suggest that phage-mediated acquisition and transfer of *gtr* operons is ongoing. Acquisition is likely to be restricted by O-antigen substrate, plausibly because the O-antigen is a known (co)-receptor for phage attachment [27]. This and the availability of a suitable genomic integration site will influence the *gtr* diversity in these bacterial genomes.

### O-antigen modification and the GtrC families

*gtr* functionality has previously been identified for single representatives of GtrC families I [8], [10], III [7] and VIII [11]. Ascribing function to additional GtrC families is an important step in understanding the biological contribution of *gtr* to *Salmonella*. Thus, as a first step we determined whether the occurrence of LPS modification could be shown for *gtr* families present in serovars *S.* Typhimurium (KW serogroup B; GtrC families III and IV), *S.* Infantis (KW serogroup C_1_; GtrC families IV, V and VI) and *S.* Typhi (KW serogroup D_1_; GtrC families II and III).

**Figure 5:**
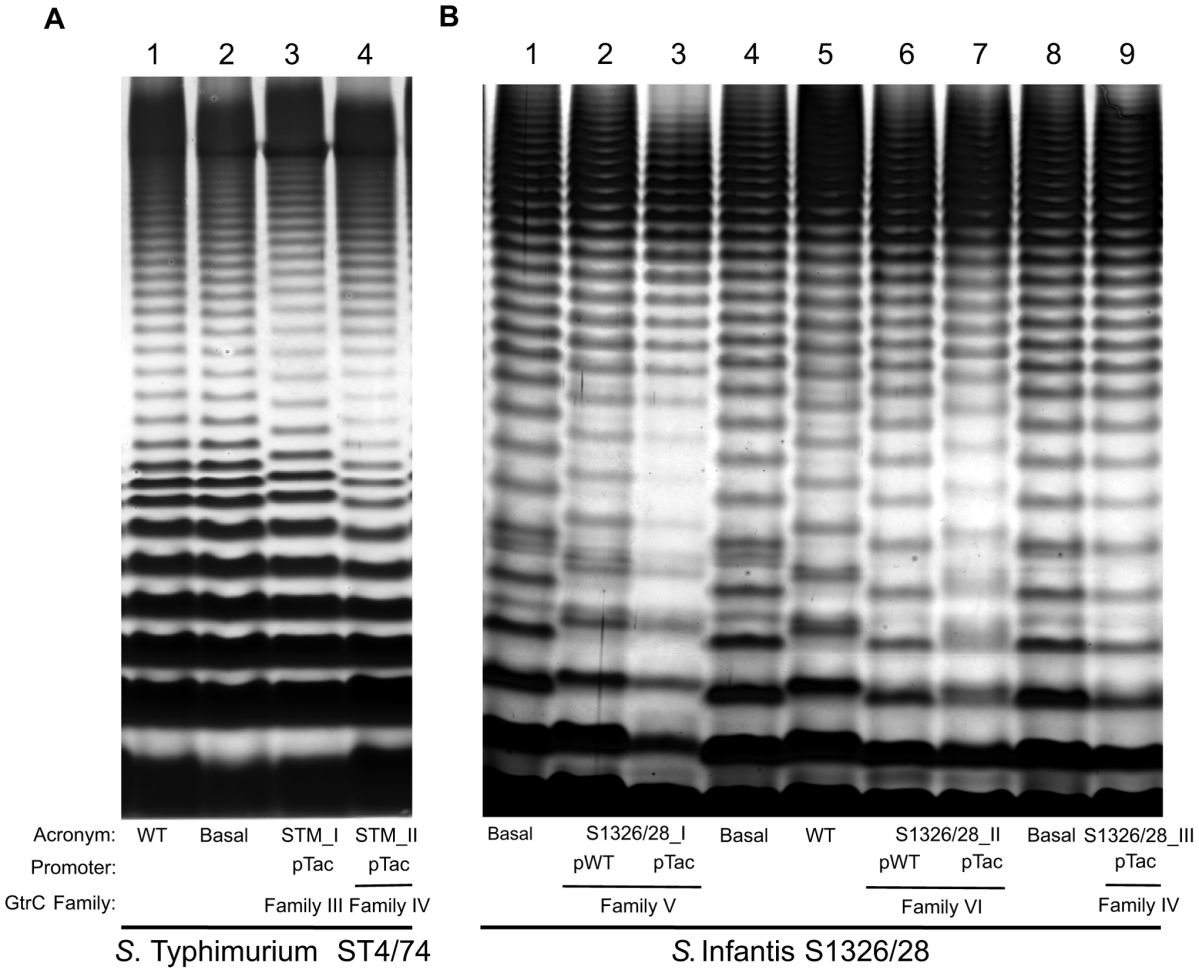
Lipopolysaccharide profiling of four GtrC families from two*S. enterica* subspecies I serovars. A) *S.* Typhimurium strain ST4/74 lipopolysaccharide (LPS) comparing the banding profiles of wildtype (WT) LPS to LPS from an ST4/74 strain devoid of all known LPS modification genes termed ‘basal’ (delta *gtr* ^STM_I^, delta *gtr* ^STM_II^ and delta *oafA*); and mutant strains expressing a single *gtr* operon *gtr* ^STM_I^ (GtrC family III) and *gtr* ^STM_II^ (GtrC family IV) driven by its native promotor, pWT, or the constitutive promoter pTac. B) *S.* Infantis strain S1326/28 LPS profiles of GtrC family V and family VI operons in comparison to LPS profiles from WT and basal (delta *gtr* ^S1326/28_I^, *gtr* ^S1326/28_II^ and *gtr* ^S1326/28_III^) *S.* Infantis strains. LPS was extracted from overnight bacterial cultures and visualized on silver stained TSDS-PAGE gels. A change in the size of LPS banding patterns is used to infer functionality of *gtr* operons.

*Salmonella* strains were constructed that were devoid of all *gtr*-like operons, which are referred to further as ‘basal’ strains [20]. As we have previously shown, phase variation can occur for some *gtr* operons [20]. Therefore, strains were also constructed containing a single *gtr* operon expressed from a well-characterized, constitutive promoter to ensure consistent expression [20], [28]. LPS banding profiles from wild type and these mutant strains were compared and a shift in the LPS ladder rungs was used as indicator of O-antigen modification and GtrC functionality (Figure 5).

Modification mediated by family III and family IV GtrC was assessed from the representative operons present in the *S.* Typhimurium strain ST4/74. Consistent with our description of phase variation of the *gtr* ^STM_I^ operon and the described bias to the OFF phase [20], no detectable change in the size of the LPS rungs is observed comparing basal and wild type LPS (Figure 5A, compare lanes 1 and 2). However, a shift is observed when the *gtr* ^STM_I^ operon is expressed from the constitutive promoter (Figure 5A, compare lanes 3 and 2). These data are consistent with the recent report that this locus mediates the addition of glucose to the galactose moiety of the *S.* Typhimurium O-antigen via an alpha 1–4 linkage (locus STM0557 - STM0559 [7]). In contrast, even when ensuring constitutive chromosomal expression of the *gtr* ^STM_II^ operon, representing GtrC family IV, no visible LPS size differences were found in comparison to the ‘basal’ STM strain (Figure 5A, compare lanes 4 and 2).

Similar to the findings of family IV, we were not able to demonstrate visible shifts as a result of expression of family II GtrC in *S.* Typhi strain BRD948 (data not shown). We think it unlikely that this lack of visible modification is due to transcriptional regulation as the promoter is a constitutive promoter, but we cannot rule out post-transcriptional regulation. A more likely explanation is that for these family II and family IV operons the modification does not yield a detectable shift in this assay, or that the enzymatic target or substrate is absent. The former could be due to either the nature of the moiety added or if *gtr* mediates a change in sugar linkage.

Finally, the functionality of three *gtr* operons in *S.* Infantis strain S1326/28 was examined. While sharing the GtrC family IV with KW serogroup B and C_2_ serovars, this serogroup C_1_ serovar also harbors two KW serogroup C_1_ specific *gtr* operons, family V (*gtr* ^S1326/28_I^) and family VI (*gtr* ^S1326/28_I1^) (Figure 1). The LPS ladder pattern from a WT strain showed clear differences compared to that of the basal mutant (Figure 5B, compare lane 4 and 5), indicating at least one *gtr* operon mediated modification. This was apparently not due to the *S.* Infantis GtrC family IV (*gtr* ^S1326/28_III^) as no shift was observed even when it was constitutively expressed (Figure 5B, compare lanes 8 and 9). The lack of visible modification by *S.* Infantis GtrC family IV and the *S.* Typhimurium family IV GtrC (Figure 5A, lanes 1, 2) is therefore unlikely to be due to KW serogroup context. In contrast, expression of the other two families mediated *S.* Infantis LPS shifts. Family V Gtr (*gtr* ^S1326/28_I^) when expressed from both the native promoter (pWT) and constitutive promoter (pTac) resulted in visible modification (Figure 5B, compare lanes 1 to 3), whereas for GtrC family VI (*gtr* ^S1326/28_I1^), modification was only detectable when constitutively expressed (Figure 5B, compare lanes 6 to 8). The latter indicates that some *gtr* operons may not be expressed under all conditions, either due to phase variation or environmental control. Thus, for the first time we show detectable functionality for GtrC family V and family VI. Overall, of the six GtrC families examined, detectable functionality has now been shown for four GtrC families (I, III, V, VI).

### Modified LPS and changes in*Salmonella* O-serotype

The structure of the O-antigen is inherently linked with the O-serotype used in the current Kaufmann-White *Salmonella* classification scheme. Thus, *gtr*-dependent modifications may alter the outcome of O-serotyping according to clinical diagnostic practices. Using the strains described above, we determined whether *S.* Infantis O-antigens modified by GtrC family V (*gtr* ^S1326/28_I^) and family VI (*gtr* ^S1326/28_II^) (Figure 5B, lanes 3 and 7) alter the *S.* Infantis strain serotype in comparison to the WT and *gtr* negative ‘basal’ strains (Figure 5B, lanes 4 and 5). All four strains were O-serotyped by a *Salmonella* reference laboratory and were determined to be O:6,7 positive, consistent with the O:6,7 designation in the KW scheme for serogroup C_1_ serovars. Additional agglutination analyses with O:14 and O:6_1_ sera, two factors associated with O:6,7 sero-converting phage, did not yield a positive agglutination test in any of the four strains. Thus, the family V and VI *gtr* –dependent modification of the O-antigen within KW serogroup C_1_ observed here (Figure 5B) is not identified by current serotyping protocols. These findings highlight the limitations of sero-diagnostic methodologies in differentiating between sero-converting *Salmonella* isolates that have modified their immunodominant O-antigen.

### Dam-dependent phase variable control of*gtr* expression is widespread but not universal

We recently determined that expression of three *gtr* operons representing GtrC families I, II, and III is controlled by phase variation resulting in heterogeneous expression of the operon in a genetically clonal population [20]. This phase variation mechanism is epigenetic and involves Dam, a DNA methyltransferase, and OxyR a transcriptional regulator. Signature sequence elements at the *gtr* operon that are required for this regulation were identified as four Dam target sequences (GATC) and two overlapping OxyR binding motifs that each contain two GATC sequences [20].

**Figure 6:**
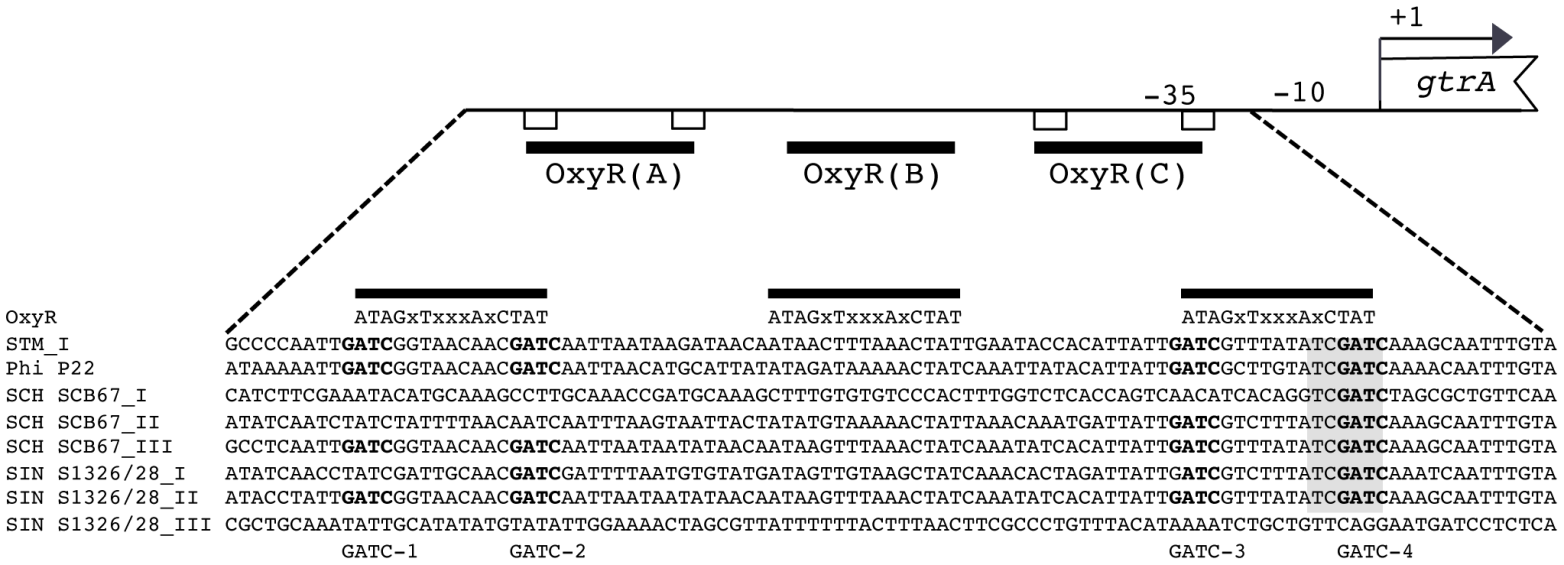
Regulatory regions from 8*gtr* operons illustrating degrees of conservation and variation in *gtr* phase variation sequence elements [**20**] **.** Top of the figure is a schematic representation of the phase variable *gtr* regulatory region showing the location of three OxyR dimer binding sequences (thick black line), GATC sequences (open rectangle), −35 and −10 promoter sequences, transcription start site (+1) and the *gtrA* open reading frame. The presence of these elements is a signature of *gtr* phase variation. Below the schematic is an alignment of seven *gtr* regulatory region sequences showing consensus OxyR dimer binding sequence, GATC sequences in bold and the −35 of the promoter shaded in grey. Sequence “SIN S1326/28_III” is shown as comparison to illustrate lack of phase variation sequence elements. This sequence was aligned based on conserved sequence surrounding the +1 site (not shown). Acronyms represented: STM, *S.* Typhimurium; SCH, *S.* Choleraesuis; SIN, *S.* Infantis. Figure S1 shows the alignment and conserved nucleotides of 128 bp of regulatory sequence from 43 *gtr* operons.

The conservation of this phase variable promoter architecture was examined by analysis of the sequence 128 bases upstream of the *gtrA* transcription start site, as previously defined [29]. Based on the 59 operons analyzed here, 43 operons distributed among 8 of the 10 GtrC families (GtrC families I, II, III, V, VI, VII, IX and X) had recognizable signature sequences suggestive of phase variation (Figure S1). Of these 43, 37 had all 4 GATC sites and OxyR-like motifs. The signature sequences of specific GtrC family III, V and VI *gtr* operons however contained various sequence variations, including some of the *gtr* operons analyzed for O-antigen modification described above (Figure 6). For example, *S.* Infantis *gtr* ^S1326/28_I^ lacked GATC-1, *S.* Paratyphi C *gtr* ^RKS4594_I^ lacked both GATC-1 and GATC-2 and *S.* Choleraesuis *gtr* ^SCB67_I^ contained only the similar -35 (encompassing GATC-4) and -10 promoter sequences. Of the 59 operons, 16 lacked sequence resembling the phase variation signature sequence, representing all GtrC family IV members and the phage epsilon34 encoded *gtr* (family VIII).

To confirm the sequence-based prediction of phase variation, transcriptional regulation was examined using a single copy, chromosomal reporter fusion (*gtr-lacZ*). Phase variation is determined by the occurrence of both *lacZ* expressing (Lac+/“On”) and non-expressing (Lac−/“Off”) colonies [29]. As predicted, based on the presence of the signature sequence, *S.* Infantis *gtr* ^S1326/28_II^ (family VI) and *S.* Choleraesuis *gtr* ^SCB67_III^ (family V) phase varied (Table 1, Figure 6). Consistent with the lack of the signatures sequences, expression of *S.* Choleraesuis *gtr* ^SCB67_I^, *gtr* ^SCB67_II^ and *gtr* ^SCB67_IV^ did not phase vary (Table 1, Figure 6).

**Table 1:** Determination of phase variable expression of*S. enterica* subspecies I Gtr families.

Strain	*gtr* regulatory region	Phase variation detected	ON – OFF Switch frequency	OFF – ON Switch frequency	Miller Units per 100% where applicable (S.D)
sMV444	*S.* Infantis	Yes	6.0×10^−3^	5.3×10^−3^	932 (27)
	*gtr* ^S1326/28_I^		6.8×10^−3^	7.6×10^−3^	
sMV445	*S.* Infantis	Yes	2.4×10^−3^	2.8×10^−3^	1225 (97)
	*gtr* ^S1326/28_II^		1.9×10^−3^	2.7×10^−3^	
sMV446	*S.* Infantis	No	N/A	N/A	177 (8)
	*gtr* ^S1326/28_III^				
sMV84	*S.* Typhimurium	Yes	2.1×10^−2^	2.0×10^−3^	1236 (40)
	*gtr* ^LT2_I^		2.2×10^−2^	1.7×10^−3^	
sMV510	*S.* Typhimurium	Yes	2.1×10^−2^	3.4×10^−3^	1346 (71)
	*gtr* ^LT2_I^ CATC-1		2.3×10^−2^	3.4×10^−3^	
sMV83*	*S.* Typhimurium	Yes	1.7×10^−3^	4.2×10^−3^	824 (74)
			1.4×10^−3^	3.7×10^−3^	
sMV511	*S.* Typhimurium	No	N/A	N/A	865 (40)
	*gtr* ^P22^ CATC-1				
sMV517	*S.* Choleraesuis	No	N/A	N/A	N/D
	*gtr* ^SCB67_I^				
sMV518	*S.* Choleraesuis	No	N/A	N/A	N/D
	*gtr* ^SCB67_II^				
sMV520	*S.* Choleraesuis	Yes	N/D	N/D	N/D
	*gtr* ^SCB67_III^				
sMV521	*S.* Choleraesuis	No	N/A	N/A	N/D
	*gtr* ^SCB67_IV^				

N/A: not applicable due to absence of detectable phase variation.

N/D: not determined.

* Data taken from [20].

To address the impact of the rare mutations that were identified at some *gtr* operons, a similar analysis was carried out for *S.* Infantis *gtr* ^S1326/28_I^ (family V) with a mutated GATC-1 sequence. This sequence still allowed for phase variation with similar switch frequencies as *gtr* operons with an intact GATC-1 sequence (Table 1; [20]). To improve the predictive power of sequence analysis for this regulation, a mutation was introduced into GATC-1 (GATC-1 to CATC-1) for two known phase varying *gtr* operons, *S.* Typhimurium *gtr* ^LT2_I^ and *gtr* ^P22^ [20]. Interestingly, the mutations did not abrogate phase variation in context of the *gtr* ^LT2_I^ sequence (sMV510), but abrogated phase variation in context of the *gtr* ^P22^ sequence (sMV511, Table 1). This analysis shows that the effect of point mutations in the signature sequence on *gtr* expression will depend on the sequence context.

Taken together, our experimental data combined with sequence analyses shows that the presence of the Dam/OxyR regulatory sequence elements can be used as a signature for *Salmonella* phase variation. Our findings highlight that not only are the makeup and complement of *gtr* operons fluid and variable within the *Salmonella* species, but there is an additional layer of complexity introduced by phase variation in determining the O-antigen phenotype in salmonellae.

## Discussion

Horizontally acquired genes appear to play a key role in defining O-serovar identity, and retention of O-antigen modification genes such as *gtr* operons by salmonellae may convey significant biological advantages. In this study, we address the frequency, sequence diversity, functionality and regulatory characteristics of *gtr* operons in salmonellae.

Through BLAST approaches of 22 complete *S. enterica* subsp. *enterica* (subspecies I) genome sequences we identified 52 *gtr* operons. Each of the subspecies I genomes examined possessed between 1 and 4 *gtr* operons. It should be noted that the *S. enterica* genomes screened were of serovars representing Kaufmann-White serogroups A-D_1_, representing 7 of the 46 KW O-serogroups. Thus, the specific number of GtrC families identified here is not highly relevant, as we predict that more GtrC families will be identified as genomes from serovars of more diverse KW O-serogroups become available. The identification of 3 *gtr* operons from the draft genomes of *S. bongori* serovars from KW O-serogroups G and R also shows that *gtr* operons are not limited to O-serogroups A - D_1_ and are not restricted to *S. enterica* subspecies I serovars.

Phylogenetic and clustering analyses of the 55 *gtr* operons with an additional 4 *gtr*-like operons derived from publically available *Salmonella* phage genome sequences, identified 10 GtrC ‘families’ (designated GtrC families I to X). Of these, three GtrC families include strains of only a single Kaufmann-White O-serogroup, serogroup C_1_ and C_2_ (Figure 1, GtrC families V, VI and VII). This may indicate that these *gtr* operons target a moiety in the O-antigen that is specific to KW O-serogroup C_1_ and C_2_ serovars. In contrast, the GtrC families (I–IV) reside within genomes from multiple serogroups. This can be understood by considering the biochemical structure of the basal (un-modified) O-antigen. For example, serogroups A, B and D_1_ all share the same basic tri-saccharide basal O-unit [Gal-Rha-Man], but differ in their side 3,6 di-deoxyhexose sugars, namely paratose, abequose and tyvelose respectively (reviewed in [26]). We found that these three serogroups may harbor GtrC family I and III members, which can mediate addition of glucose to the galactose moiety via 1–6 (O:1 serotype) [8], [10] and 1–4 linkages (O:12_2_) [7], respectively. We predict that the Gtr family III will have the same modifying activity in all serogroup A, B and D_1_ strains, as they all possess the required alpha galactose receptor (ie. O:12 positive). This prediction is supported by the observation that family I and family III GtrC operons were not identified in serogroup C_1_ genomes, which have a basal O-unit that lacks the galactose moiety [26]. In these genomes, we identified the serogroup C_1_ unique GtrC families V, VI and VII. In contrast, GtrC family II and family IV were not confined to serogroups with similar O-antigen biochemical structures. Interestingly, we did not find evidence that the O-antigen is a substrate for these two GtrC families based on our LPS gel-based assay and no activity has been assigned to any members of those two families. Collectively, these analyses highlight that the O-antigen structure is a defining factor for the repertoire of GtrC families that any one serovar can possess.

O-antigen modifying operons appear to be associated with bacteriophage genomes that use the O-antigen as their receptor. In fact, all 16 known O-antigen modification operons in *Shigella flexneri* are associated with mobile elements [13], [30]. In *Salmonella*, *gtrABC*-like genes are present on at least 3 different P22-like temperate phage genomes [11]. Our analyses support these observations and extend the presence of *gtr* operons across the *Salmonella* genus. Our genome analyses placed all *Salmonella gtr* operons within four previously defined ‘regions of difference’ that were purported to be of phage origin [11], [25]. Consistent with these studies, we found that two of these regions, locus 1 (P22 locus) and locus 2 (SPI-16 locus) contain *gtrABC* operons within full-length prophage sequences (Figure 3). Locus 2 appears to be a hot-spot for *gtr* insertion with all *gtr*-positive serovars harboring *gtr* in this loci (Figure 3) and some strains containing multiple *gtr* operons (Figure 4). The identification of GtrC families and KW O-serogroup associations is consistent with the observation that phage are drivers of *gtr* dissemination, and as such, phage can only lysogenize when their O-antigen structural receptor is present.

Not all *gtr*-dependent modification is reflected in the current KW scheme. For example, we demonstrated that the apparent modification due to expression of GtrC families V and VI in the KW serogroup C_1_ serovar *S.* Infantis (Figure 5B, lanes 3 and 7) did not alter the O-serotype based on standard clinical typing practices or known serogroup C_1_ subtype sera (O:6_1_). Although not completely unexpected as serotyping is based on a limited number of sera and the KW scheme eliminates many known phage based variations including some known *gtr* modifications [1], the findings reiterate that KW O-serogroups are more heterogeneous regarding the composition of expressed surface structures than previously thought. Therefore, the currently applied KW O-serotyping scheme and assignment of O-serogroups does not and cannot differentiate the extensive diversity in O-antigen repertoire. This is in contrast to the situation with *Shigella flexneri*, where phage associated *gtr* dependent modification has been retained in the serotyping scheme [13]. Further functional characterization of the *gtr* families is ongoing, and should provide additional insight into antigenic variation that exists among *Salmonella* serovars.

The homology between any two GtrA and GtrB clusters was high, >84% and >90% amino acid similarity respectively. In contrast, inter-family GtrC homology was low, <46% similarity. This sequence variation between GtrC families is unlikely to be the result of high diversifying selection but suggests that either they perform distinct functions or they have convergently evolved to perform the same or related functions. Given the lack of sequence conservation, evolutionary linkages between GtrC families could not be inferred, as there is little genomic evidence that they share a common ancestor. Similar observations of sequence diversity have been observed for the GtrC-like O-antigen modification genes of *Shigella* [13], and in general, significant sequence divergence is not unusual among functionally related glycosyltransferases [31]. The lack of substantial sequence divergence for GtrA and GtrB is consistent with the *Shigella* counterparts [13]. Our clustering and recombination analyses identified a mosaic of gene shuffling events leading to new allelic variants of *gtr* gene sequences. Furthermore, our analysis of KW serogroup C_1_ genomes is suggestive of gene rearrangements occurring between chromosomal and phage-associated *gtr* operons resulting in ‘new’ combinations of *gtrABC* families. The generation of sequence diversity could be facilitated by lysogenization of a strain containing one or more *gtr* operons by phage with a complimentary *gtr* repertoire. This would also suggest that evolutionary history of the *gtr* operon is dynamic and ongoing which in turn blur any phylogenetic inferences.

The presence of premature stop codons in three GtrC gene products (*S.* Paratyphi A, GtrC family III and *S.* Choleraesuis, family IV) suggests that not all GtrC families contain functional products. The presence of these rare *gtrC* pseudogenes may be indicative of recent selection such as immune pressure. Similarly, the *gtrB* gene from GtrC family II shares a conserved deletion, yet the *gtrC* in all genomes remain intact. All family II GtrC genomes harbor a second *gtr* operon, which raises the possibility that this may enable functionality of the family II GtrC gene product. Alternatively, GtrB may not be a functional requirement for this operon. Support for the former possibility comes from analyses in *Shigella* showing that at least some GtrA and GtrB can be used interchangeably without affecting GtrC function [32], and our finding of extensive recombination within *Salmonella gtrAB* gene sequences that are not restricted by gene boundaries. Thus, there may a degree of redundancy in the Gtr system, but further studies are required to confirm this. The presence of multiple *gtr* operons and the general lack of *gtrC* mutations within *S. enterica* subspecies I serovars suggest a selective pressure to retain *gtrC*-dependent activity, which in turn promotes O-antigen diversity.

In addition to diversity in the repertoire and sequences of GtrC families within *Salmonella* serovars, we previously found that clonal heterogeneity may arise through regulation of *gtr* expression by phase variation [20]. Here we describe further evidence that phase variation is widespread among most GtrC families irrespective of *Salmonella* species, subspecies or serotype. Experimentally verified phase variation correlated with the presence of signature sequences for OxyR- and Dam-dependent regulation [20]. The loss of one of the four Dam target sequences in the signature may abrogate phase variation, however, this is dependent on the sequence context. This likely is a result of the specific OxyR binding sequence yielding different OxyR binding affinity, which in turn will impact the epigenetic regulation [20], [33]. The absence or presence of this characterized phase variation mechanism may still allow input from additional factors on *gtr* expression, even though we are yet to find growth conditions that significantly alter expression level, or induce or eliminate phase variation (unpublished). The relevance of the apparent lack of phase variation in specific *gtr* families and individual operons is not clear at present but as *gtr*-specific modifications and roles are elucidated, we may gain further insight. Interestingly, there is no evidence suggesting that phase variation controls expression of the *Shigella flexneri gtr* operon that has been shown to have a role in virulence [34]. We furthermore propose that phase variable *gtr* expression contributes to the variability in biochemical glucosylation observed in some O-antigen NMR studies (reviewed in [26], [7]). This in turn underpins the weak and variable agglutination reactions that can be reported from diagnostic laboratories, and that are represented by brackets in the Kaufmann-White *Salmonella* classification scheme [1].

The large and variant repertoire of *gtr* operons in and among *Salmonella* serovars shows that there is an additional layer of LPS heterogeneity that is largely not being detected by current serotyping methodologies. The incorporation of phase variable elements into *gtr* expression promotes further complexity onto the structure of the immunodominant O-antigen. Full understanding of the impact of *gtr* expression will require comprehensive analyses that take into consideration the biochemistry of modification, serovar context and the related host and virulence variables. Based on the current insight, it is likely that these *gtr*-mediated strain differences are important for understanding *Salmonella* virulence, persistence and spread, and based on findings with *Shigella* [35] could impact on vaccine development. Taken together, utilizing the extensive occurrence and significant sequence variation between stochastically expressed GtrC families could aid in developing higher resolution molecular serotyping methods [36] for *Salmonella* diagnostics.

## Materials and Methods

### Identification of*gtr* operons within *Salmonella* genome sequences

In order to examine the distribution of *gtr* operons, 24 publically available finished *Salmonella* genomes sequences were obtained from the European Bioinformatics Institute (listed in Table S1). To address *gtr* presence in non-subspecies I *S. enterica* serovars four draft genomes from The Wellcome Trust Sanger Institute (http://www.sanger.ac.uk/cgi-bin/blast/submitblast/salmonella) or The Genome Institute at Washington University (http://genome.wustl.edu/tools/blast) were screened using BLAST. Furthermore, draft genome sequences of 28 *S. bongori* genomes comprising of 8 different KW O-serogroups were also analyzed (Table S2, [37]). Four publically available *Salmonella* bacteriophage genome sequences that are known to encode for *gtr* operons, specifically Phi P22, Phi ST64T, Phi DT104 and epsilon 34, were also included [9]–[11], [38], [39].

The presence of *gtr* genes in complete genome sequences was identified in two ways. The *gtrC* from bacteriophage P22 was used to search *Salmonella* genome sequences using BLASTn. Hits were considered to be *gtr* if the *gtrC* homolog sequence had neighboring *gtrB* and/or *gtrA*-like sequences. All identified *gtrA, gtrB* and *gtrC* genes were placed in a sequence file against which genome sequences were re-analyzed by BLASTn to confirm preliminary findings. Second, genomes representing the same KW serogroup were aligned using the Artemis Comparison Tool (ACT) [40] to examine site-specific integration of *gtr* operons. This also enabled characterization of regions flanking *gtr* operons for signatures of mobile genetic elements such as transposable elements or phage-like sequences. Examination of trans-membrane topology of the Gtr gene products were predicted using a hidden Markov model, TMHMM 2.0 [41].

### Nomenclature

For consistency, we adopt the O-antigen modifying glycosyltransferase (*gtr*) nomenclature used in our previous study [20], which is a modification of that proposed previously [13]. Putative *gtr*-like operons identified through BLAST analyses were numerically assigned based on their genomic location in relation to the purported *oriC*. For example, *S.* Typhimurium *gtr* ^LT2_I^ represents the ‘first’ *gtr* operon (relative to the *oriC*) in the genome of *S.* Typhimurium strain LT2. Roman numeral designations were assigned based on ascending gene number and was employed to avoid conflict with strain numbering. Assigned acronyms are summarized in Table S1. This descriptive *gtr* gene nomenclature precisely defines the serovar and strain, and thus, is beneficial given the large variation in *gtr* repertoire within and among strains and serovars as identified in this study. It can be expanded upon without loss of clarity.

### Multiple sequence alignment, phylogenetic, clustering and recombination analyses

To determine the relationship of the GtrA, GtrB and GtrC gene products, phylogenetic analyses were performed on aligned sequences. All sequence alignments were carried out on both nucleotide and translated nucleotide sequences for accuracy. Automated alignments using ClustalW2 [42] were manually adjusted to account for differences in the size of *gtr* gene operons. The genome sequences represented in this study are biased towards serovars representing significant mammalian pathovars. For example, *S.* Typimurium is represented by 6 genome sequences. All conserved *gtr* operons identified within *S.* Typhimurium were identical when aligned. Thus, in order to reduce unnecessary over-representation of identical sequences, the *gtr* operons from serovar *S.* Typhimurium strains LT2, 14028s, SL1344, D23580, DT2 and DT104 are represented by one single acronym ‘STM’. As all *S.* Typhimurium genomes examined in this study contain a minimum of two conserved *gtr*-like operons, the final number of *gtr* operons used in phylogenetic analyses was reduced from 58 to 48. Phylogenetic trees were constructed for GtrA, GtrB and GtrC using both maximum likelihood and baysian interference methods. Maximum likelihood trees were estimated in PhyML using optimal WAG model for protein and GTR model for nucleotide sequences with rate heterogeneity estimated from the data. All other options were default. One thousand non-parametric bootstraps were applied for robustness.

To examine the clustering of the *gtr* genes, we performed a statistical genetic analysis using the software package Bayesian Analysis of Population Structure, BAPS [23], [24]. Genetic clusters were designated using a nucleotide sequence alignment for the *gtrA*, *gtrB* and *gtrC* genes. BAPS clusters sequences based on shared polymorphisms using a non-reversible stochastic optimization algorithm that identifies the number of clusters that best fits the dataset. As GtrC is purported to convey serotype-specific modifications, the *gtrC* gene was used to define *gtr* clusters, designated in this study as GtrC families.

To examine the role of recombination within the evolutionary history of the *gtr* operon, homoplasic SNPs were identified for each branch of *gtrA* and *gtrB* phylogenetic trees and mapped onto concatenated *gtrAB* sequences using PAML [43]. Clusters of matching homoplasies located along different branches of the phylogenetic tree were used as markers of recombination.

### Bacterial strains and culture conditions

In order to examine O-antigen modifying activity of the *gtr* operons, *Salmonella enterica* subsp. *enterica* serovars Infantis (*S.* Infantis) strain S1326/28 and Typhimurium (*S.* Typhimurium) strain ST4/74 were used. All strains were a kind gift from Dr Rob Kingsley, The Wellcome Trust Sanger Institute, UK. Bacteria were routinely cultured aerobically at 37°C in Luria-Bertani (LB) broth or on LB with 1.75% agar. Strains harboring the temperature sensitive plasmids pKD46 and pSIM18 and were grown aerobically at 30°C. Where required, cultured strains carrying antibiotic resistant markers were supplemented with antibiotics (Sigma) at the following concentrations: ampicillin, 100 µg ml^−1^; kanamycin, 30 µg ml^−1^; hygromycin, 100 µg ml^−1^ and tetracycline, 12.5 µg ml^−1^.

### Generation of*gtr* mutants in *Salmonella*

*S.* Typhimurium and *S.* Infantis with defined mutations were constructed using the Lambda-Red recombination system [44], [45]. *gtr* specific oligonucleotides were designed for site specific deletion of *gtr* operons through amplification of a kanamycin marker (*kan*) from plasmid pKD13 [44] or the tetracycline (*tetRA*) cassette from transposon Tn*10d*Tc [46]. Primer sequences are shown in Table S3. Confirmation of integration and allelic exchange was performed by PCR as described previously [44], [45] or by DNA sequencing.

For construction of strains with multiple mutations in chromosomal genes, the *tetRA* cassette was replaced with an oligonucleotide (Table S3) using the Lambda-Red approach in combination with counter-selection for loss of tetracycline resistance [46], [47] to generate in-frame deletions on the chromosome in the absence of a genetic scar. This same counter-selection procedure was employed to generate constitutive expression of *gtr* operons. Specifically, 154 bp of *S.* Infantis *gtr* ^S1326/28_I^; 174 bp *gtr* ^S1326/28_II^ and 263 bp of *gtr* ^S1326/28_III^ upstream of the assigned *gtrA* start codon were first replaced with *tetRA*, after which *tetRA* was replaced with 116 bp containing the pTac promoter [28] derived from pMAL-c2x (New England Biolabs). pTac is a constitutive promoter in *Salmonella* since they do not encode the *lac* operon and repressor. Expression of *gtr* was confirmed by reverse transcriptase PCR or by LPS gel analyses.

### LPS extraction

Crude LPS extracts were prepared from 3 ml overnight bacterial LB cultures grown with aeration at 37°C. Cultures were pelleted by centrifugation at 3 700 *g* for 15 min at 4°C (Sorvall) and washed three times with 0.9% NaCl. Cells were resuspended in 125 µl ice-cold buffer solution (60 mM Tris-HCL, 1 mM EDTA, pH 6.8) containing 2% SDS and boiled for 5 mins. Lysed cells were diluted in 875 µl of buffer solution without SDS. Enzymatic digestion of nucleic acids was performed using RNase (Roche) and DNase (Promega) for four hours at 37°C, followed by proteinase K (100 µg) treatment overnight at 50°C. The crude LPS preparations were stored at 4°C for analysis.

### Tricine-sodium dodecyl sulfate-polyacrylamide gel electrophoresis (TSDS-PAGE) analysis and silver staining

For analyses of LPS profiles from *Salmonella* mutants, crude LPS preparations were analysed on 20 cm×15 cm; 10.5% separating, 4% stacking TSDS-PAGE gels as modified from [48]. An equal volume of 2× sample buffer (6% sodium dodecyl sulfate; 6% 2-mercaptoethanol; 10 mM dithiothreitol; 46% glycerol; 60 mM Tris-HCl, pH 8.0; 0.1% bromophenol blue) was added to the crude LPS preparations and boiled for 5 min. LPS was visualized by oxidative silver staining, modified from [49]. Briefly, gels were fixed overnight in fixative solution (30% ethanol; 10% acetic acid) then oxidized with 0.7% periodic acid in fixative solution for 10 mins. Gels were washed 3×15 min in ddH_2_O before staining with 0.1% silver nitrate for 30 mins. Gels were washed briefly in excess ddH_2_O before developing in a 3% sodium carbonate, 0.02% formaldehyde solution until desired staining intensities were achieved.

### Analysis of the*gtr* phase varion

The approach and methods used to analyze *gtr* phase variation were performed as described [20] using a CRIM-based [50], chromosomal, transcriptional fusion to *lacZ* in an *S.* Infantis S1326/28 or *S.* Typhimurium LT2 background. Primer sequences are shown in Table S3. Expression levels were determined using the β-galactosidase assay [51] and, where relevant, correction for % On in the culture was introduced by calculating and expressing the Miller units per 100% “On”.

### Serotypic analysis of*gtr* mutants

*Salmonella* mutants were serotyped by routine clinical diagnostic procedures at the Scottish *Salmonella*, *Shigella* and *Clostridium difficile* Reference Laboratory, Glasgow, where standard serum agglutination assays were performed using purified O-antigen serum routinely used for diagnostic procedures. Sub-type O:6_1_ specific serum was a kind gift from Dr Peter Roggentin (Institute for Hygiene and Environment, Hamburg) and was used in-house in a standard agglutination assay.

## Supporting Information

Figure S1 Regulatory region sequence alignments from 43 *gtr* operons harboring sequence elements similar to that of the known phase variable *gtr* ^LT2_I^ regulon (GtrC family III) [20]. 128 base pair regions upstream of the putative +1 transcriptional start site [29] were aligned using ClustalW2. Asterisks indicate conserved residues. Three putative OxyR binding sites are shown and the Dam methyltransferase recognition sequences (GATC) are represented in bold font and underlined as defined in [20]. The +1 transcription start site and −35 and −10 promoter sequences [29] are indicated in grey shaded regions. The phase variable promoter architecture is conserved across most *gtr* regulatory regions. Strain acronyms are defined in Table S1.
(PDF)

Table S1 Genome coordinates of 52 *gtr* operons identified in 22 *Salmonella enterica* subsp. *enterica* (subspecies I) serovar genome sequences.
(PDF)

Table S2 *S. bongori* isolates^+^ screened for the presence of *S. enterica*-like *gtr* operons.
(PDF)

Table S3 Primer sequences used for lambda red mutagenesis and for generation of *gtr* regulatory region-*lacZ* reporter fusion constructs.
(PDF)
