# Supplementary material for: Horizontally Acquired Glycosyltransferase Operons Drive Salmonellae Lipopolysaccharide Diversity
Source: PLoS Genet. 2013 Jun 20;9(6):e1003568. doi: 10.1371/journal.pgen.1003568 (PMC3688519; doi:10.1371/journal.pgen.1003568)
Supplement: Table S1 — Genome coordinates of 52 gtr operons identified in 22 Salmonella enterica subsp. enterica (subspecies I) serovar genome sequences. (PDF) [file pgen.1003568.s002.pdf]

Table S1.

S. enterica serovar and strain^	Kaufmann-White Serogroup [$]	Genome accession number	*gtr* genome coordinates (operon)	*gtr* genome orientation	Genome systematic ID's +	Assigned Gtr operon acronym[@]
Paratyphi A ATCC_9150	Group A	CP000026	540234-540923	gtrCB*A	SPA 0469- 0467	9150_ I
			2256846-2259761	gtrABC*	SPA 2167- 2169*	9150_II
			2486780-2489515	gtrABC	SPA 2385-2387	9150_III
Paratyphi A AKU_12601	Group A	FM200053	538409-540957	gtrCB*A	SSPA 0434- 0432	12601_I
			2252030-2254945	gtrABC*	SSPA 2016- SSPA 2017a	12601_II
			2482316-2485051	gtrABC	SSPA 2224- 2226	12601_III
Typhimurium LT2	Group B	AE006468	614807-617722	gtrCBA	STM 0559- 0557	LT2_I
			4424449-4427281	gtrCBA	STM 4206-4204	LT2_II
Typhimurium 14028s	Group B	CP001363	615501-618416	gtrCBA	STM14 0650-0652	14028s_I
			4437988-4440820	gtrCBA	STM14 5056-5054	14028s_II
Typhimurium DT104	Group B	N/A	403911-406646	gtrCBA	SDT 0361- 0363	DT104_I
			638657-641572	gtrCBA	SDT 0583- 0585	DT104_II
			4501354-4504186	gtrCBA	SDT 4190- 4192	DT104_III
Typhimurium D23580	Group B	FN424405	406441-408710	gtrCA*	STMMW 03911-03921	D23580_BTP1

			653614-656532	gtrCBA	STMMW 06231-06251	D23580_II
			4447157-4449989	gtrCBA	STMMW 41531-41551	D23580_III
Typhimurium SL1344	Group B	FQ312003	614295-617210	gtrCBA	SL 0547- SL0549	SL1344_I
			4445738-4448570	gtrCBA	SL 4139- 4141	SL1344_II
Typhimurium DT2	Group B	N/A	614791-617706	gtrCBA	DT2 05511-05491	DT2_I
			4382759-4385591	gtrCBA	DT2 40551-40531	DT2_II
Heidelberg SL476	Group B	CP001120	413031-415766	gtrABC	SeHA C0414-0416	SL476_I
			663237-666152	gtrCBA	SeHA C0669-0667	SL476_II
			4420397-4423229	gtrCBA	SeHA C4543-C4541	SL476_III
Schwarzengrund CVM19633	Group B	CP001127	699407-700284	gtrCBA	SeSA A0725-A0723	CVM19633
Agona SL483	Group B	CP001138	597653-600568	gtrCBA	SeAg B0604-0602	SL483_I
			4344080-4346912	gtrCBA	SeAg B4459-4457	SL483_II
Paratyphi B SPB7	Group B	CP000886	2500338-2503252	gtrABC	SPAB 03002-03004	SPB7
Choleraesuis SC-B67	Group C_1	AE017220	410262-412636	gtrCBA	SCH 0368- 0370	SCB67_I
			660997-663862	gtrABC	SCH 0592- 0594	SCB67_II
			665196-667922	gtrCBA	SCH 0595- 0597	SCB67_III
			4359067-4361899	gtrC*BA	SCH PS147*-4085	SCB67_IV

Infantis S1326/28	Group C_1	N/A	623726-626452	gtrABC	SIN 05331-05351	S1326/28_I
			627786-630651	gtrCBA	SIN 05381-05401	S1326/28_II
			4312816-4315648	gtrCBA	SIN 38151-38171	S1326/28_III
Paratyphi C RKS4594	Group C_1	CP000857	609844-612471	gtrABC	SPC 0569- 0571	RKS4594_I
			614418-616768	gtrCBA	SPC 0578- 0576	RKS4594_II
			4354486-4357318	gtrCBA	SPC 4266 -4264	RKS4594_III
Newport SL254	Group C_2	CP001113	619047-622131	gtrCBA	SNSL254 A0609-0607	SL254_I
			4415618-4418450	gtrCBA	SNSL254 A4544-4542	SL254_II
Hadar 18	Group C_2	N/A	628402-631489	gtrCBA	SHAD 05371-05391	SHAD18_I
			4366513-4369345	gtrCBA	SHAD 38781-38801	SHAD18_II
Typhi TY2	Group D_1	AE014613	540204-542752	gtrCB*A	t0468- 0466	TY2_I
			2371423-2374338	gtrABC	t2303- 2305	TY2_II
Typhi CT18	Group D_1	AL513382	606763-609678	gtrCBA	STY 0607- 0605	CT18_I
			2461960-2464508	gtrAB*C	STY 2627a- 2629	CT18_II
Enteritidis P125109	Group D_1	AM933172	594265-597180	gtrCBA	SEN 0537- 0535	P125109_I
			2501054-2503603	gtrABC	SEN 2376- 2378	P125109_II
Gallinarum 287/91	Group D_1	AM933173	614286-615553	gtrCBA	SG 0569- 0567	287/91_I

			2491925-2494474	gtrABC	SG 2427- 2429	287/91_II
Dublin CT_02021853	Group D_1	CP001144	655577-658312	gtrCBA	SeD A0656- 0654	CT02021853_I
			659799-662714	gtrCBA	SeD A0660- 0658	CT02021853_II
			2628156-2630705	gtrABC	SeD A2750- 2751	CT02021853_III

FOOTNOTES:

N/A: Not available. Genome sequences are freely available for BLAST analyses (http://www.sanger.ac.uk/cgi-bin/blast/submitblast/salmonella)

^ An additional 34 genome sequences representing non-*S. enterica* subsp. *enterica* serovars (subspecies I) were analyzed: The genomes constitute the complete genome of *S. bongori* strain 12419 (accession number FR877557) and 28 draft *S. bongori* strains representing 8 different O-serotypes (Table S2, [37]); *S. enterica* subsp. *salamae* (subspecies II); *S. enterica* subsp. *arizonae* str. RSK2980 (accession CP000880-subspecies IIIa); *S. enterica* subsp. *diarizonae* str. CDC 01-0005 (subspecies IIIb); *S. enterica* subsp. *houtenae* (subspecies IV); *S. enterica* subsp. *indica* (subspecies V). Draft genome sequences are publically available from The Wellcome Trust Sanger Institute or The Genome Institute at Washington University.

+ As assigned by original publication.

* Contain a possible frameshift mutation or deletion that may render the product non-functional.

[$] According to the Kauffman-White scheme [1], [2].

[@] In order to reduce unnecessary over-representation of identical sequences, the *gtr* operons from the six *S*. Typhimurium (STM) strains represented in this study (LT2, 14028s, SL1344, D23580, DT2 and DT104) were annotated collectively as STM+_I and STM+_II in Figure 1.
