# Supplementary material for: Horizontally Acquired Glycosyltransferase Operons Drive Salmonellae Lipopolysaccharide Diversity
Source: PLoS Genet. 2013 Jun 20;9(6):e1003568. doi: 10.1371/journal.pgen.1003568 (PMC3688519; doi:10.1371/journal.pgen.1003568)
Supplement: Table S2 — S. bongori isolates+ screened for the presence of S. enterica-like gtr operons. (PDF) [file pgen.1003568.s003.pdf]

Table S2.

Strain Name	SRA sample submission*	Kaufmann-White[$] serotype	Kaufmann-White[$] serogroup	*gtr* operon present (Y/N)
RKI-1593	ERS004192	O66:z65:-	O:66	N
CDC-2703-76	ERS004191	O48:z41:-	Y (O:48)	N
IP7688/91	ERS002039	O66:z39:-	O:66	N
285 CT	ERS004193	O48:z35:-	Y (O:48)	N
CEIM 44847	ERS004249	O48:z35:-	Y (O:48)	N
CEIM 44833	ERS004196	O48:z35:-	Y (O:48)	N
RKI-1373	ERS004176	O60:z41:-	O:60	N
12419 (ATCC 43975)	EMBL accession no. FR877557	O66:z35:-	O:66	N
RKI-1397	ERS004190	O66:z35:-	O:66	N
RKI-1398	ERS004173	O66:z65:-	O:66	N
IP1900/76	ERS004246	O66:z35:-	O:66	N
RKI-1786	ERS004174	O66:z81:-	O:66	N
RKI-1300	ERS004170	O48:z35:-	Y (O:48)	N
CEIM 46049	ERS004175	O48:z35:-	Y (O:48)	N
IP6031/93	ERS002041	O13,22:r:-	G (O:13)	N
IP0224/66	ERS002029	O48:z39:-	Y (O:48)	N

IP1387/73	ERS002030	O1,40:z81:-	R (O:40)	Y
IP2022/77	ERS002031	O44:r:-	V (O:44)	N
IP3846/83	ERS002032	O44:z39:-	V (O:44)	N
IP4327/84	ERS002033	O1,40:z35:-	R (O:40)	Y
IP6794/89	ERS002038	O48:b:-	Y (O:48)	N
IP4504/84	ERS002034	O48:z81:-	Y (O:48)	N
IP4770/84	ERS002035	O1,13,22:i:-	G (O:13)	Y
IP4985/85	ERS002036	O48:z39:-	Y (O:48)	N
IP5713/87	ERS002037	O61:z35:-	O:61	N
IP7804/92	ERS002040	O48:z65:-	Y (O:48)	N
IP9325/02	ERS002044	O13,22:z39:-	G (O:13)	N
IP8365/95	ERS002042	O48:i:-	Y (O:48)	N

+ *S. bongori* genome sequences as defined by [37].

* SRA: Short read archive. Available for download at the European Nucleotide Archive http://www.ebi.ac.uk/ena/

$ Kaufmann-White refers to the Kaufmann-White-Le Minor *Salmonella* classification scheme [1], [2].
