# Supplementary material for: Horizontally Acquired Glycosyltransferase Operons Drive Salmonellae Lipopolysaccharide Diversity
Source: PLoS Genet. 2013 Jun 20;9(6):e1003568. doi: 10.1371/journal.pgen.1003568 (PMC3688519; doi:10.1371/journal.pgen.1003568)
Supplement: Figure S1 — Regulatory region sequence alignments from 43 gtr operons harboring sequence elements similar to that of the known phase variable gtr LT2_I regulon (GtrC family III) [20]. 128 base pair regions upstream of the putative +1 transcriptional start site [29] were aligned using ClustalW2. Asterisks indicate conserved residues. Three putative OxyR binding sites are shown and the Dam methyltransferase recognition sequences (GATC) are represented in bold font and underlined as defined in [20]. The +1 transcription start site and −35 and −10 promoter sequences [29] are indicated in grey shaded regions. The phase variable promoter architecture is conserved across most gtr regulatory regions. Strain acronyms are defined in Table S1. (PDF) [file pgen.1003568.s001.pdf]

```
                                   OxyR(A)                         OxyR(B)                         OxyR(C)
                                ATAGxTxxxAxCTAT                 ATAGxTxxxAxCTAT                 ATAGxTxxxAxCTAT
                                **   *   * * **                 ***        ****                 ** *     *   *
                               GATC-1       GATC-2                                             GATC-3       GATC-4
                                ***  *   * * *** **             ***     *  ****   *      ***********    ****** **    ******** *  ** *** **** ***** ***
SeHA SL476_II        -GCCCCAATTGATCGGTAACAACGATCAATTAATAAGATAACAATAACTTTAAACTATTGAATACCACATTATTGATCGTTTATATCGATCAAAGCAATTTGTAGTGCTACACTCCAGACCTTTCCGAA
SEN P125109_I        -GCCCCAATTGATCGGTAACAACGATCAATTAATAAGATAACAATAACTTTAAACTATTGAATACCACATTATTGATCGTTTATATCGATCAAAGCAATTTGTAGTGCTACACTCCAGACCTTTCCGAA
STM LT2_I            -GCCCCAATTGATCGGTAACAACGATCAATTAATAAGATAACAATAACTTTAAACTATTGAATACCACATTATTGATCGTTTATATCGATCAAAGCAATTTGTAGTGCTACACTCCAGACCTTTCCGAA
STM D23580_II        -GCCCCAATTGATCGGTAACAACGATCAATTAATAAGATAACAATAACTTTAAACTATTGAATACCACATTATTGATCGTTTATATCGATCAAAGAAATTTGTAGTGCTACACTCCAGACCTTTCCGAA
STM DT104_II         -GCCCCAATTGATCGGTAACAACGATCAATTAATAAGATAACAATAACTTTAAACTATTGAATACCACATTATTGATCGTTTATATCGATCAAAGCAATTTGTAGTGCTACACTCCAGACCTTTCCGAA
STM 14028_I          -GCCCCAATTGATCGGTAACAACGATCAATTAATAAGATAACAATAACTTTAAACTATTGAATACCACATTATTGATCGTTTATATCGATCAAAGCAATTTGTAGTGCTACACTCCAGACCTTTCCGAA
STM DT2_I            -GCCCCAATTGATCGGTAACAACGATCAATTAATAAGATAACAATAACTTTAAACTATTGAATACCACATTATTGATCGTTTATATCGATCAAAGCAATTTGTAGTGCTACACTCCAGACCTTTCCGAA
STM SL1344_I         -GCCCCAATTGATCGGTAACAACGATCAATTAATAAGATAACAATAACTTTAAACTATTGAATACCACATTATTGATCGTTTATATCGATCAAAGCAATTTGTAGTGCTACACTCCAGACCTTTCCGAA
SG 287/91_I          -GCCCCAATTGATCGGTAACAACGATCAATTAATAAGATAACAATAACTTTAAACTATTGAATACCACATTATTGATCGTTTATATCGATAAAAGCAATTTGTAGTGCTACACTCCAGACCTTTCCGAA
SeSa CVM19633        -GCCCCAATTGATCGGTAACAACGATCAATTAATAAGATAACAATAAGTTTAAACTATCAAATATCACATTATTGATCGTTTATATCGATCAAAGCAATTTGTAGTGCTACACTCCAGACCTTTCCGAA
STY CT18_I           -GCTCCAATTGATCGGTAACAACGATCAATTAATAAGACAACAATAAGTTCAAGCTATCAAATATAACATTATTGATCGTTTATATCGAAAAAAGCAATTTGTAGTACTATACTCCAGACCTTTCCGAA
STY TY2_II           -GCTCCAATTGATCGGTAACAACGATCAATTAATAAGACAACAATAAGTTCAAGCTATCAAATATAACATTATTGATCGTTTATATCGAAAAAAGCAATTTGTAGTACTATACTCCAGACCTTTCCGAA
SPB                  -GCCTCAATCGATCGATAACAACGATCAATTAATAATATAACAATAAGTTTAAACTATCAAATATCACATTATTGATCGTTTATATCGATCAAATCAATTTGTAGTGCTACACTCCAGACCTTTCCGAA
SN SL254_I           -GCCTCAATTGATCGGTAACAACGATCAATTAATAATATAACAATAAGTTTAAACTATCAAATATCACATTATTGATCGTTTATATCGATCAAATCAATTTGTAGTGCTACACTCCAGACCTTTCCGAA
SeAg SL483_I         -GCCTCAATTGATCGGTAACAACGATCAATTAATAACATAACAATAAGTTTAAACTATCAAATATCACATTATTGATCGTTTATATCGATCAAATCAATTTGTAGTGCTACACTCCAGACCTTTCCGAA
SCH SCB67_III        -GCCTCAATTGATCGGTAACAACGATCAATTAATAATATAACAATAAGTTTAAACTATCAAATATCACATTATTGATCGTTTATATCGATCAAAGCAATTTGTAGTGCTACACTCCAGACCTTTCCGAA
SPC RKS4594_II       -GCCTCAATTGATCGGTAACAACGATCAATTAATAATATAACAATAAGTTTAAACTATCAAATATCACATTATTGATCGTTTATATCGATCAAAGCAATTTGTAGTGCTACACTCCAGACCTTTCCGAA
SIN S1326/28_II      -ATACCTATTGATCGGTAACAACGATCAATTAATAATATAACAATAAGTTTAAACTATCAAATATCACATTATTGATCGTTTATATCGATCAAAGCAATTTGTAGTGTTACACTCCAGACCTTTCCGAA
SHAD 18_I            -ATACCTATTGATCGGTAACAACGATCAATTAATAATATAACAATAAGTTTAAACTATCAAATATCACATTATTGATCGTTTATATCGATCAAATCAATTTGTAGTGCTACACTCCAGACCTTTCCGAA
Phi ST104            -ATAAAAATTGATCGGTAACAACGATCAATTAACGAGCAAACAATAACTTTAAACTATCAAAGTTTACATTATTGATCGTTTATATCGATCAAAGTAATTTGTAGTGCTACACTCCAGACCTTTCCGAA
STM DT104_I          -ATAAAAATTGATCGGTAACAACGATCAATTAACGAGCAAACAATAACTTTAAACTATCAAAGTTTACATTATTGATCGTTTATATCGATCAAAGTAATTTGTAGTGCTACACTCCAGACCTTTCCGAA
Phi ST64T            -ATAAAAATTGATCGGTAACAACGATCAATTAACGAGCAAACAATAACTTTAAACTATCAAAGTTTACATTATTGATCGTTTATATCGATCAAAGCAATTTGTAGTGCTACACTCCAGACCTTTCCGAA
SPA 9150_II          -ATAAAAATTGATCGGTAACAACGATCAATTAACGCGCAAACAATAACTTTAAACTATCAAAGATTACATTATTGATCGTTTATATCGATCAAGGCAATTTGTAGTGCTACACTCCAGACCTTTCCGAA
SPA 9150_III         -ATAAAAATTGATCGGTAACAACGATCAATTAACGCGCAAACAATAACTTTAAACTATCAAAGATTACATTATTGATCGTTTATATCGATCAAGGCAATTTGTAGTGCTACACTCCAGACCTTTCCGAA
SPA 12601_II         -ATAAAAATTGATCGGTAACAACGATCAATTAACGCGCAAACAATAACTTTAAACTATCAAAGATTACATTATTGATCGTTTATATCGATCAAGGCAATTTGTAGTGCTACACTCCAGACCTTTCCGAA
SPA 12601_III        -ATAAAAATTGATCGGTAACAACGATCAATTAACGCGCAAACAATAACTTTAAACTATCAAAGATTACATTATTGATCGTTTATATCGATCAAGGCAATTTGTAGTGCTACACTCCAGACCTTTCCGAA
SeD CT02021853_I     -ATACCTATTGATCGGTAACAGCGATCAATTAACGAGCAAACAATAACTTTAAACTATCAAAGTTTACATTATTGATCGTTTATATCGATCAAAGCAATTTGTAGTGCTACACTCCAGACCTTTCCGAA
SeD CT02021853_II    -ATACCTATTGATCGGTAACAGCGATCAATTAACGAGCAAACAATAACTTTAAACTATCAAAGTTTACATTATTGATCGTTTATATCGATCAAAGCAATTTGTAGTGCTACACTCCAGACCTTTCCGAA
SPA 9150_I           -ATACCAATTGATCGATAATATCGATCAATATATTAAAACTCAATAGCTTAAAACTATTAAAAATACAATTATTGATCGCTTATATCGATCAAACCAATTTGTAGTGCTACACTCCAGACCTTTCTGAA
SPA 12601_I          -ATACCAATTGATCGATAATATCGATCAATATATTAAAACTCAATAGCTTAAAACTATTAAAAATACAATTATTGATCGCTTATATCGATCAAACCAATTTGTAGTGCTACACTCCAGACCTTTCTGAA
STY CT18_II          -ATACCAATTGATCGATAACATCGATCAATATATTAAAACTCAATAGCTTAAAACTATTAAAAATACAATTATTGATCGCTTATATCGATCAAACCAATTTGTAGTGCTACACTCCAGACCTTTCTGAA
STY TY2_I            -ATACCAATTGATCGATAACATCGATCAATATATTAAAACTCAATAGCTTAAAACTATTAAAAATACAATTATTGATCGCTTATATCGATCAAACCAATTTGTAGTGCTACACTCCAGACCTTTCTGAA
SEN P125109_II       -ATACCAATTGATCGATAACATCGATCAATATATTAAAACTCAATAGTTTAAAACTATTAAAAATACAATTATTGATCGCTTATATCGATCAAACCAATTTGTAGTGCTACACTCCAGACCTTTCTGAA
SG 287/91_II         -ATACCAATTGATCGATAACATCGATCAATATATTAAAACTCAATAGTTTAAAACTATTAAAAATACAATTATTGATCGCTTATATCGATCAAACCAATTTGTAGTGCTACACTCCAGACCTTTCTGAA
SeDu CT02021853_III  -ATACCAATTGATCGATAACATCGATCAATATATTAAAACTCAATAGTTAAAAACTATTAAAAATACAATTATTGATCGCTTATATCGATCAAACCAATTTGTAGTGCTACACTCCAGACCTTTCTGAA
SCH SCB67_II         -ATATCAATCTATCTATTTTAACAATCAATTTAAGTAATTACTATATGTAAAAACTATTAAACAAATGATTATTGATCGTCTTTATCGATCAAAGCAATTTGTAGTGCTACACTCCAGACCTTTCCGAA
SPC RKS4594_I        -ATATCAATCTATCTATTTTAACAATCAATTTAAGTAATTACTATATGTAAAAACTATTAAACAAATGATTATTGATCGTCTTTATCGATCAAAGCAATTTGTAGTGCTACACTCCAGACCTTTCCGAA
SIN S1326/28_I       -ATATCAACCTATCGATTGCAACGATCGATTTTAATGTGTATGATAGTTGTAAGCTATCAAACACTAGATTATTGATCGTCTTTATCGATCAAATCAATTTGTAGTGCTACACTCCAGACCTTTCCGAA
Phi P22              -ATAAAAATTGATCGGTAACAACGATCAATTAACATGCATTATATAGATAAAAACTATCAAATTATACATTATTGATCGCTTGTATCGATCAAAACAATTTGTAGTGCTACACTTCAGATCTTTCCGAA
SeHA SL476_I         -ATAAAAATTGATCGGTAACAACGATCAATTAACATGCATTATATAGATAAAAACTATCAAATTATACATTATTGATCGCTTGTATCGATCAAAACAATTTGTAGTGCTACACTTCAGACCTTTCCGAA
S. bongori IP1387/73 -CTAAAGATTGATCGATATCAACGATCAATCAACCATTTCATAATAGCTATAAACTATCTTATATTGCATTATTGATCGTTAATATCGATCAAGACAATTTGTAATGCTACACTTCAGACCTTTCTGAA
S. bongori IP4327/84 -CTAAAGATTGATCGATATTAACGATCAATCAACCATTTCATAATAGCTATAAACTATCTTATATTGCATTATTGATCGTTAATATCGATCAAGACAATTTGTAATGCTACACTTCAGACCTTTCTGAA
S. bongori IP4770/84 AATAAAG-CTGATCGATATTAACGATCAATTGACCATTTTATAATAGGCAAATACTATCTTATATCACATTATTGATCGTTAATATCGATCAAGCCAATTTGTAATGCTACACTTCAGACCTTTCTGAA
                                                                                                           -35                    -10       +1
```

Figure S1.
