# Supplementary material for: Horizontally Acquired Glycosyltransferase Operons Drive Salmonellae Lipopolysaccharide Diversity
Source: PLoS Genet. 2013 Jun 20;9(6):e1003568. doi: 10.1371/journal.pgen.1003568 (PMC3688519; doi:10.1371/journal.pgen.1003568)
Supplement: Table S3 — Primer sequences used for lambda red mutagenesis and for generation of gtr regulatory region-lacZ reporter fusion constructs. (PDF) [file pgen.1003568.s004.pdf]

Table S3.

Primer name	Primer sequence (5' – 3')	Description
	**Primer sequences used for lambda red mutagenesis***	
oMV442kan	TATTTCCGTAATATTCTCATTTGTCCTCGCCCCTGTTCTAACGTCCCATGTgtgtaggctggagctgcttc	*gtrC* primer for allelic replacement of *S*. Infantis *gtr*S1326/28_III and *S*. Typhimurium *gtr*STM_II (GtrC Family IV) with kanamycin.
oMV444kan	ATTAGTCCCTTTTCGCGCTATTTCCGATGAAAATGTAATCACTTTGCGattccggggatccgtcgacc	*gtr* regulatory region primer for allelic replacement of *S*. Infantis *gtr*S1326/28_III and *S*. Typhimurium *gtr*STM_II (GtrC Family IV) with kanamycin.
oMV728tetR	GACCTTTCCGAATCCGCTGATTTTCATAATGTTGAAGTTATTCGCTttaagacccactttcacatt	*gtr* regulatory region primer for allelic replacement of *S*. Infantis *gtr*S1326/28_I (GtrC Family V) with tetracycline.
oMV730tetA	GAATTATGGACAGGCTCCTTGTTGACTACTCTATAACAATCCTTGCctaagcacttgtctcctg	*gtrC* primer for allelic replacement of *S*. Infantis *gtr*S1326/28_I (GtrC Family V) with tetracycline.
oMV731	AGGCTCCTTGTTGACTACTCTATAACAATCCTTGCAGCGAATAACTTCAACATTATGAAAATCAGCGGAT	Replacement of tetRA cassette from *S*. Infantis *gtr*S1326/28_I::tetRA (GtrC Family V)
oMV735tetR	ATTACAGTCTCTGAGGCGGAATTATAGTTAATTACACCACTTAGGttaagacccactttcacatt	*gtrC* primer for allelic replacement of *S*. Infantis *gtr*S1326/28_II (GtrC Family VI) with tetracycline.
oMV737tetA	AACATGCAAAGCCTTGCAAACCAATGCAAAGCTTTGTGTGTCCCGctaagcacttgtctcctg	*gtr* regulatory region primer for allelic replacement of *S*. Infantis *gtr*S1326/28_II (GtrC Family VI) with tetracycline.
oMV739	GCCTTGCAAACCAATGCAAAGCTTTGTGTGTCCCGCCTAAGTGGTGTAATTAACTATAATTCCGCCTCAG	Replacement of tetRA cassette from *S*. Infantis *gtr*S1326/28_II::tetRA (GtrC Family V)

oMV526tetR	GAACACCGATCGATGTGTACTTAGCGAATAACTTCAACATttaagacccactttcacatt	*gtrA* primer for allelic replacement of *S*. Infantis *gtr*S1326/28_II regulatory region (GtrC Family VI) with tetracycline
oMV528pTac	GAACACCGATCGATGTGTACTTAGCGAATAACTTCAACATgactggctgtttcctgtg	*gtrA* primer for allelic replacement of *S*. Infantis *gtr*S1326/28_II regulatory::tetRA (GtrC Family VI) with the pTac promoter.
oMV753pTac	AACATGCAAAGCCTTGCAAACCAATGCAAAGCTTTGTGTGTCCCGgacatcataacggttctggc	*gtr* regulatory region primer for allelic replacement of *S*. Infantis *gtr*S1326/28_II regulatory::tetRA (GtrC Family VI) with the pTac promoter.
oMV531tetR	GCAGTATTAAGTACGCCTATCGAAACGTACTTTATAAATAATTTTATCATttaagacccactttcacatt	*gtrA* primer for allelic replacement of *S*. Infantis *gtr*S1326/28_III regulatory region and *S*. Typhimurium *gtr*STM_II (GtrC Family IV) with tetracycline.
oMV532tetA	GCTTCGCCTTATCCAGCCTGCAAAAGGTGCATAAACACCGctaagcacttgtctcctg	*gtr* regulatory region primer for allelic replacement of *S*. Infantis *gtr*S1326/28_III and *S*. Typhimurium *gtr*STM_II regulatory region (GtrC Family IV) with tetracycline.
oMV533pTac	GCAGTATTAAGTACGCCTATCGAAACGTACTTTATAAATAATTTTATCATgactggctgtttcctgtg	*gtrA* primer for allelic replacement of *S*. Infantis *gtr*S1326/28_III *S*. Typhimurium *gtr*STM_II regulatory::tetRA (GtrC Family IV) with the pTac promoter.
oMV534pTac	GCTTCGCCTTATCCAGCCTGCAAAAGGTGCATAAACACCGgacatcataacggttctggc	*gtr* regulatory region primer for allelic replacement of *S*. Infantis *gtr*S1326/28_III *S*. Typhimurium *gtr*STM_II regulatory::tetRA (GtrC Family IV) with the pTac promoter.
oMV764tetR	GACTCGATATAAATGGGCTAAGAGCTTTTGCATTGATAAGTGTTGTGCTGttaagacccactttcacatt	*oafA* 5’ primer for allelic replacement of *oafA* with tetracycline.
oMV765tetA	CCTGAGCCTTCTGGTGTTAGGTGCGCATTGTCATATTGTATAGGGTAAGCctaagcacttgtctcctg	*oafA* 3’ primer for allelic replacement of *oafA* with tetracycline.

oMV766	GGCTAAGAGCTTTTGCATTGATAAGTGTTGTGCTGG CTTACCCTATACAATATGACAATGCGCACCTAAC	*oafA* primer for allelic replacement of *oafA*:tetRA.
oMV496tetR	TATTTCCGTAATATTCTCATTTGTCCTCGCCCCTGTT CTAACGTCCCATGTttaagacccactttcacatt	*gtrC* primer for allelic replacement of *S*. Typhimurium *gtr*STM_I (GtrC Family III) with tetracycline.
oMV497tetA	TTGTCCCAAACTACTTAGCAATCAGTAGCCCCAATT GATCGGTAACAACGctaagcacttgtctcctg	*gtr* regulatory region primer for allelic replacement of *S*. Typhimurium *gtr*STM_I (GtrC Family III) with tetracycline.
oMV498	CTCATTTGTCCTCGCCCCTGTTCTAACGTCCCATGTC GTTGTTACCGATCAATTGGGGCTACTGATTGCTA	Replacement of tetRA cassette from *S*. Typhimurium *gtr*STM_I::tetRA (GtrC Family III)
oMV877tetR	AATTGCTGTAATTTCACCTTTGTCTACAGAAGCGTA GTACCAGAATTCATttaagacccactttcacatt	*gtrC* primer for allelic replacement of *S*. Infantis *gtr*S1326/28_III and *S*. Typhimurium *gtr*STM_II (GtrC Family IV) with tetracycline.
oMV877tetA	ATTAGTCCCTTTTCGCGCGCTATTTCCGATGAAAAT GTAATCACTTTGCGctaagcacttgtctcctg	*gtr* regulatory region primer for allelic replacement of *S*. Infantis *gtr*S1326/28_III and *S*. Typhimurium *gtr*STM_II (GtrC Family IV) with tetracycline.
oMV878	ACCTTTGTCTACAGAAGCGTAGTACCAGAATTCATC GCAAAGTGATTACATTTTCATCGGAAATAGCGC	Replacement of tetRA cassette from *S*. Infantis *gtr*S1326/28_III::tetRA and *S*. Typhimurium *gtr*STM_II::tetRA (GtrC Family IV).
oMV439kan	TATTTCCGTAATATTCTCATTTGTCCTCGCCCCTGTT CTAACGTCCCATGTgtgtaggctggagctgcttc	*gtrC* primer for allelic replacement of *S*. Typhimurium *gtr*STM_II (GtrC Family III) with kanamycin.
oMV441kan	TTGTCCCAAACTACTTAGCAATCAGTAGCCCCAATT GATCGGTAACAACGattccggggatccgtcgacc	*gtr* regulatory region primer for allelic replacement of *S*. Typhimurium *gtr*STM_II (GtrC Family III) with kanamycin.
oMV526tetR	GAACACCGATCGATGTGTACTTAGCGAATAACTTC AACATttaagacccactttcacatt	*gtrA* primer for allelic replacement of *S*. Typhimurium STM_II regulatory region (GtrC Family III) with tetracycline.

oMV527tetA	CTTCGAAAAACATGCAAAGCCTTGCAAGCCGATGC AAAGCctaagcacttgtctcctg	*gtr* regulatory region primer for allelic replacement of *S*. Typhimurium *gtr*STM_II (GtrC Family III) with tetracycline.
oMV528pTac	GAACACCGATCGATGTGTACTTAGCGAATAACTTC AACATgactggctgtttcctgtg	*gtrA* primer for allelic replacement of *S*. Typhimurium *gtr*STM_II regulatory::tetRA (GtrC Family III) with the pTac promoter.
oMV529pTac	CTTCGAAAAACATGCAAAGCCTTGCAAGCCGATGC AAAGCgacatcataacggttctggc	*gtr* regulatory region primer for allelic replacement of *S*. Typhimurium *gtr*STM_II regulatory::tetRA (GtrC Family III) with the pTac promoter.
	**Primer sequences used for cloning and mutagenesis of *gtr* regulatory region- *lacZ* reporter fusion constructs (in order of strains Table 1)****	
oMV412	catgatggtaccCTTCAACATTATGAAAATCAGCGG	R-primer regulatory region for generating *lacZ* fusion of sMV444, sMV445, sMV84, and sMV352; with Acc651 site
oMV846	ggtagctctgcagAACCCTCGACTTAGAAGTTTCTG	F-primer Regulatory region for *lacZ* fusion sMV444 with PstI site
oMV848	ggtagctctgcagCCCGCGGCTTAGATGTTCCTGG	F-primer Regulatory region for *lacZ* fusion sMV445 with PstI site
oMV458	catgatggtaccTTTTATCATTATAGCAATCA	R-primer regulatory region for generating *lacZ* fusion of sMV446 and sMV521; with Acc651 site
oMV459	ggtagctctgcagGCTTCGCCTTATCCAGCCTG	F-primer Regulatory region for *lacZ* fusion sMV446, with PstI site
oMV414	ttgattcctgcagCCCACGGCTTAGATGTTCCTGG	F-primer Regulatory region for *lacZ* fusion sMV84 and sMV520 with PstI site

oMV504	AGCAATCAGTAGCCCCAATTCATCGGTAACAACGA	Mutagenic sense primer for GATC 1 mutation in regulatory region for *lacZ* fusion in sMV510
oMV505	TCGTTGTTACCGATGAATTGGGGCTACTGATTGCT	Mutagenic antisense primer for GATC 1 mutation in regulatory region for *lacZ* fusion in sMV511sMV510
oMV514	CAAACCACTTAGCAATCAGCAATAAAAATTCATCG GTAACAACGATC	Mutagenic sense primer for GATC 1 mutation in regulatory region for *lacZ* fusion in SMV511
oMV515	GATCGTTGTTACCGATGAATTTTTATTGCTGATTGC TAAGTGGTTTG	Mutagenic antisense primer for GATC 1 mutation in regulatory region for *lacZ* fusion in sMV511
oMV413	ggtagctctgcagCTTCGCATTACGAATTATAAGAAC	F-primer Regulatory region for *lacZ* fusion sMV517 with PstI site
oMV850	catgatggtaccTGTAAACAACTATGAGTCCTTGGA	R-primer regulatory region for generating *lacZ* fusion of sMV517, with Acc651 site
oMV852	ggtagctctgcagCAATCTATCTATTTTAACAATCAATTTA AG	F-primer Regulatory region for *lacZ* fusion sMV518 with PstI site
oMV851	ggtagctctgcagAGTCCACGGCTTAGAAGTTTCTGTAACC GCCG	F-primer Regulatory region for *lacZ* fusion sMV520 with PstI site
oMV459	ggtagctctgcagGCTTCGCCTTATCCAGCCTG	F-primer Regulatory region for *lacZ* fusion sMV521 with PstI site

* Lowercase primer sequences indicate homology to the amplified antibiotic marker used for knockout selection or the pTac promoter. Synonyms: Kan, kanamycin (as designed by [44]); tetR/A, tetracycline as designed by [46]. pTac: refers to the constitutive pTac promoter (116bp) [28] that was used for allelic replacement of the native *gtr* promoter.

** Lowercase primer sequences indicate sequence added to include restriction enzyme recognition sequence.
